# Effects of *fou8/fry1* Mutation on Sulfur Metabolism: Is Decreased Internal Sulfate the Trigger of Sulfate Starvation Response?

**DOI:** 10.1371/journal.pone.0039425

**Published:** 2012-06-18

**Authors:** Bok-Rye Lee, Stine Huseby, Anna Koprivova, Aurore Chételat, Markus Wirtz, Sam T. Mugford, Emily Navid, Charles Brearley, Shikha Saha, Richard Mithen, Rüdiger Hell, Edward E. Farmer, Stanislav Kopriva

**Affiliations:** 1 John Innes Centre, Norwich Research Park, Norwich, United Kingdom; 2 Department of Plant- and Environmental Sciences, Norwegian University of Life Sciences, Aas, Norway; 3 Department of Plant Molecular Biology, University of Lausanne, Lausanne, Switzerland; 4 Heidelberg Institute for Plant Sciences (HIP), Im Neuenheimer Feld 360, Heidelberg, Germany; 5 University of East Anglia, School of Biological Sciences, Norfolk, United Kingdom; 6 Institute of Food Research, Norwich Research Park, Norwich, United Kingdom; French National Centre for Scientific Research, Université Paris-Sud, France

## Abstract

The *fou8* loss of function allele of adenosine bisphosphate phosphatase FIERY1 results in numerous phenotypes including the increased enzymatic oxygenation of fatty acids and increased jasmonate synthesis. Here we show that the mutation causes also profound alterations of sulfur metabolism. The *fou8* mutants possess lower levels of sulfated secondary compounds, glucosinolates, and accumulate the desulfo-precursors similar to previously described mutants in adenosine 5′phosphosulfate kinase. Transcript levels of genes involved in sulfate assimilation differ in *fou8* compared to wild type Col-0 plants and are similar to plants subjected to sulfate deficiency. Indeed, independent microarray analyses of various alleles of mutants in *FIERY1* showed similar patterns of gene expression as in sulfate deficient plants. This was not caused by alterations in signalling, as the *fou8* mutants contained significantly lower levels of sulfate and glutathione and, consequently, of total elemental sulfur. Analysis of mutants with altered levels of sulfate and glutathione confirmed the correlation of sulfate deficiency-like gene expression pattern with low internal sulfate but not low glutathione. The changes in sulfur metabolism in *fou8* correlated with massive increases in 3′-phosphoadenosine 5′-phosphate levels. The analysis of *fou8* thus revealed that sulfate starvation response is triggered by a decrease in internal sulfate as opposed to external sulfate availability and that the presence of desulfo-glucosinolates does not induce the glucosinolate synthesis network. However, as well as resolving these important questions on the regulation of sulfate assimilation in plants, *fou8* has also opened an array of new questions on the links between jasmonate synthesis and sulfur metabolism.

## Introduction

Arabidopsis gene At5g63980, *FIERY1*, encodes a bifunctional enzyme possessing 3′ (2′),5′-bisphosphate nucleotidase and inositol polyphosphate 1-phosphatase activities [1]. Among its *in vitro* substrates are several important cellular metabolites: inositol 1,4,5-triphosphate (IP3), which is important for the phospholipid signalling [2], 3′-phosphoadenosine 5′-phosphosulfate (PAPS), the donor of active sulfate for sulfotransferase reactions, and 3′-phosphoadenosine 5′-phosphate (PAP), which is a byproduct of these sulfotransferases [3], [4]. Therefore, it is not surprising that the gene has been identified in various genetic screens for a great range of phenotypes and that it possesses a large number of alternative names.

The Arabidopsis gene was first described as *SAL1* in a screen for plant genes increasing Li^+^ tolerance of yeast [1]. It is similar to yeast *Met22* essential for sulfate assimilation in yeast [5], which catalyses the dephosphorylation of PAPS and PAP. This gene is a target for salt toxicity in yeast and is named alternatively as *HAL2* [5]. The genetic screen that gave the Arabidopsis gene the commonly used name *FIERY1* or *FRY1* was designed to identify genes affecting abscisic acid and stress signalling [2]. Afterwards, the gene has been identified in screens for genes affecting cold signalling as *HOS2* (high expression of osmotically responsive genes) [6], for RNA silencing suppressors [7], for elevated expression of ascorbate peroxidase 2 as *ALX8* [8], for genes required for venation patterning as *RON1* [9], for mutants with deregulated fatty acid oxygenation rate as *FOU8* [10], and for mutations affecting expression of phosphate transporter [11].

**Figure 1:**
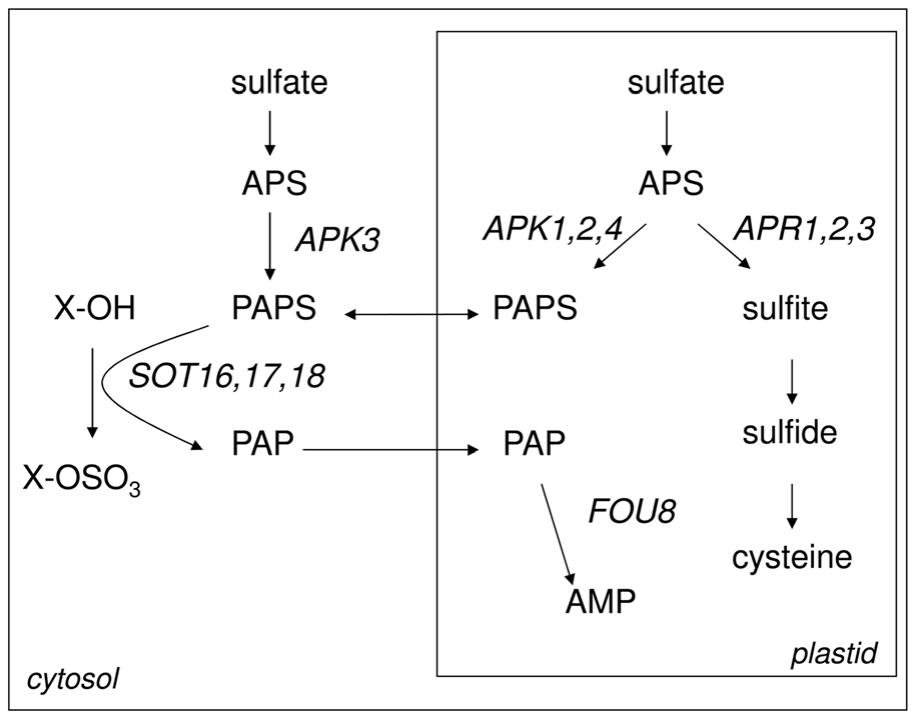
Scheme of involvement of FOU8 in sulfur metabolism.

Nearly all the different phenotypes of *fry1* mutants have been ascribed to disruptions of inositol signalling [5], [6], [9], inhibition of exoribonucleases of the XRN family by accumulated PAP [7], [11], [12], or both [8]. However, since the gene product is a metabolic enzyme, we were interested whether disruption of *FIERY1* in the *fou8* mutant would also lead to a metabolic phenotype, namely whether it would disrupt the synthesis of sulfur containing molecules like glucosinolates. The glucosinolates are a large group of sulfur-rich amino acid-derived metabolites, found mainly in the Brassicaceae [13], [14] and involved in defence against herbivores and insects, as well as fungi and bacteria [14], [15], [16]. The final step of the core glucosinolate synthesis is sulfation of the desulfo-glucosinolate precursors (Figure 1) [17]. In Arabidopsis, the sulfotransferases (SOT) of group VII, AtSOT16, 17 and 18, are responsible for this reaction [18], [19].

A close link between *FIERY1* and glucosinolates is supported by several recent reports. Apart from the substrate of FIERY1, PAP, being produced during glucosinolate synthesis, the gene has been found to be co-regulated with other genes of the pathway [20]. Also, crossing of the *fou8* mutant with *apk1 apk2*, which is characterised by low PAPS production and glucosinolate content, attenuated or prevented a range of phenotypes of the single *fou8* mutant [10]. Here we show that indeed, disruption of *FIERY1* in the *fou8* allele [10] leads to decrease in glucosinolate content with a concomitant accumulation of desulfo-glucosinolate precursors. We report a new phenotype of *fou8/fry1*, low sulfur content, and show that this is connected with changes in PAP/PAPS rather than phosphoinositols or RNA processing. Importantly, the analysis of *fou8* mutant revealed important clues to sensing and signalling in sulfur metabolism.

## Results

### Disruption of *FIERY1* inhibits glucosinolate synthesis

**Figure 2:**
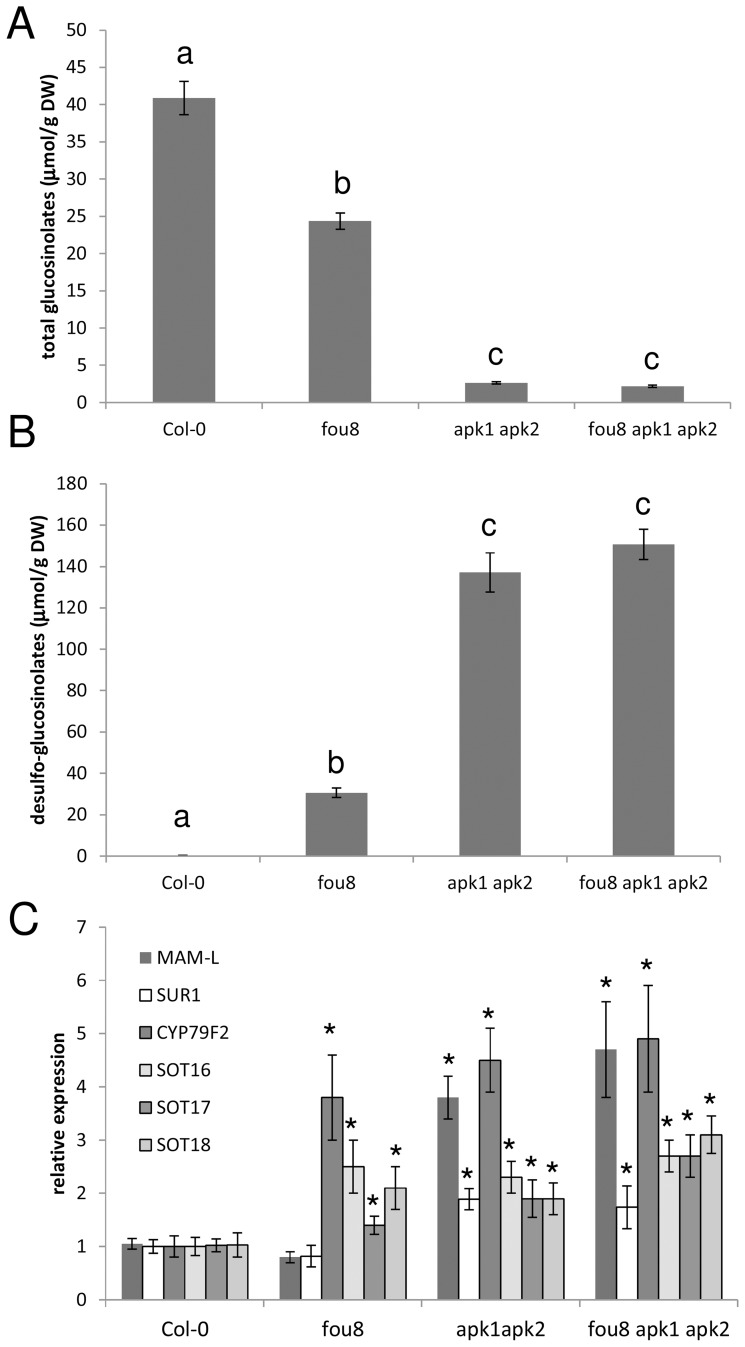
*fou8* is affected in glucosinolate synthesis. Col-0, *fou8*, *apk1 apk2*, and *fou8 apk1 apk2* plants were grown for 5 weeks in controlled environment room. The total content of **A** glucosinolates and **B** desulfo-glucosinolates was measured in leaves. **C** Total RNA was isolated from leaves and the transcript levels of six genes involved in glucosinolate synthesis was determined by quantitative RT-PCR. The qRT-PCR reactions were performed in triplicate for each biological sample. The values in Col-0 were set to 1 for all genes. Results are presented as means ± SE from six pools of three individual plants grown in two independent experiments. Different letters mark values significantly different at P<0.05; asterisks mark values significantly different from Col-0 at P<0.05.

The phenotypes caused by mutations of the *FIERY1* gene have mostly been attributed to disruption of signalling [2], [6], [8], [9]. However, as the substrate for the enzyme, PAP, is produced during synthesis of glucosinolates (Figure 1), and the gene is co-expressed with genes involved in glucosinolate synthesis [20], we tested whether disruption of *FIERY1* affects glucosinolate levels. Indeed, in the *fou8* mutant the total glucosinolate content is significantly lower than in wild type Col-0 plants (Figure 2a). As the mutation affects the last step in glucosinolate synthesis, sulfation of the desulfo-precursors, these precursors accumulate in the *fou8* mutants (Figure 2b). Similar, but more profound changes in glucosinolate contents were observed previously in the *apk1 apk2* mutant with strongly inhibited synthesis of PAPS (Figure 2) [20]. The triple mutant *fou8 apk1 apk2* has the same glucosinolate and desulfo-glucosinolate levels as *apk1 apk2*, showing that the mutations are indeed affecting the same metabolic step. Interestingly, not all individual glucosinolates were affected to the same level in *fou8*. The decrease in total glucosinolates was mostly caused by lowering the levels of aliphatic glucosinolates, while the indolic glucosinolates were affected to lesser degree or unaffected (Table 1).

**Table 1:** Levels of individual glucosinolates in Col-0 and *fou8*.

	Col-0	*fou8*	Ratio *fou8*/Col-0
4MSOB^A^	1.65±0.27	0.86±0.02	0.52
4MTB^A^	0.18±0.01	0.05±0.01	**0.27**
8MSOO^A^	0.25±0.03	0.1±0.01	0.42
4OHI3M^I^	0.018±0.007	0.015±0.002	**0.83**
I3M^I^	0.42±0.07	0.39±0.01	**0.94**
4MOI3M^I^	0.17±0.01	0.09±0.01	0.51
1MOI3M^I^	0.007±0.002	0.03±0.01	**3.83**
total	2.69±0.37	1.53±0.03	0.57

Col-0 and *fou8* plants were grown for 5 weeks in controlled environment room. Leaves were harvested and the levels of glucosinolates (µmol/g FW) were determined by HPLC. Results are presented as means ± SD from three individual plants characteristic of four independent experimental repeats. Ratios of individual glucosinolates different from the ratio of total glucosinolates by more than 25% are printed bold.

The reduced synthesis of PAPS in the *apk1 apk2* mutant resulted in coordinated increases in transcript levels of genes involved in glucosinolate synthesis (Table S1) [20]. Mugford et al. [20] concluded that this regulation is caused either by accumulation of desulfo-glucosinolates or decrease in glucosinolate levels. Since *fou8* also accumulated desulfo-glucosinolates, we tested whether they are responsible for the up-regulation, by comparing mRNA levels of several genes of the glucosinolate synthesis network in *fou8* (Figure 2c). In contrast to *apk1 apk2* and *fou8 apk1 apk2*, where transcript levels for all genes tested were up-regulated, in *fou8* the tested genes were regulated differently. From the three genes involved in synthesis of glucosinolate backbone tested, one (*CYP79F2*) was up-regulated whereas two genes (*MAM-L*, *SUR1*) were not affected. On the other hand, mRNA levels of genes responsible for the metabolic step directly affected by *fou8* mutation, sulfotransferases *SOT16*, *SOT17* and *SOT18*, were up-regulated co-ordinately. To obtain a better insight into the regulation of the glucosinolate synthesis network, we used available microarray data obtained with various alleles of *fry1/fou8* [8], [9] and compared the gene expression with microarray data of *apk1 apk2* mutant [20] (Table S1). From the 42 genes, 36 of the glucosinolate synthesis network as compiled in [21] and 6 MYB factors controlling the network [22], 33 were up-regulated at least 1.5-fold in *apk1 apk2*. In contrast, in *fry1-1* (C24 background) and *alx8* allele (Col-0) [8], nine and ten genes, respectively were up-regulated while in *ron1-1* allele (Col-0) [9] only four genes were affected. Thus, it seems that in mutants with disrupted *fiery1* gene the increase in desulfo-glucosinolates per se does not trigger the co-ordinated activation of the biosynthetic network as in *apk1 apk2* mutant.

**Figure 3:**
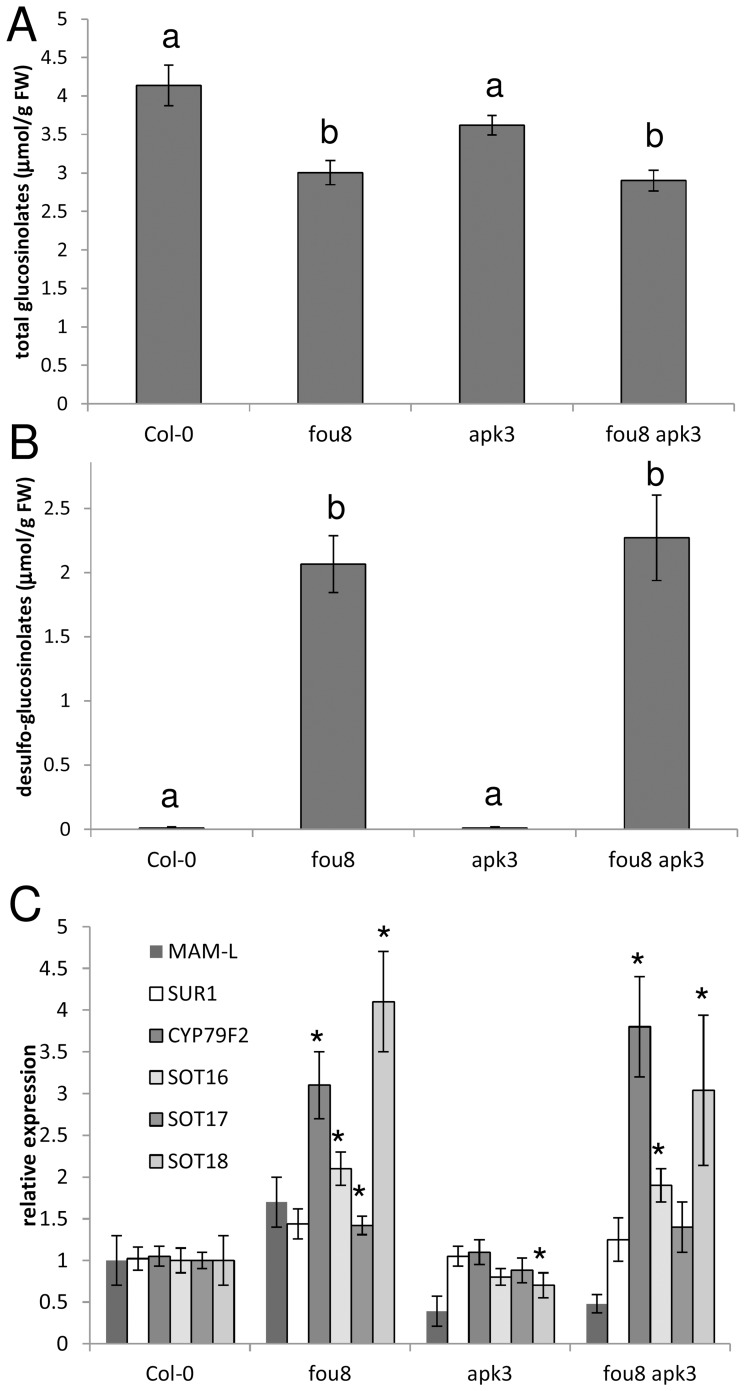
Glucosinolate synthesis in *fou8* mutant with and without cytosolic APS kinase. Col-0, *fou8*, *apk3*, and *fou8 apk3* plants were grown for 5 weeks in controlled environment room. The total content of **A** glucosinolates and **B** desulfo-glucosinolates was measured in leaves. **C** Total RNA was isolated from leaves and the transcript levels of six genes involved in glucosinolate synthesis was determined by quantitative RT-PCR. The qRT-PCR reactions were performed in triplicate for each biological sample. The values in Col-0 were set to 1 for all genes. Results are presented as means ± SE from six pools of three individual plants grown in two independent experiments. Different letters mark values significantly different at P<0.05; asterisks mark values significantly different from Col-0 at P<0.05.

Once we established that glucosinolate synthesis is affected in *fou8*, we hypothesised that this phenotype may be more pronounced by restricting PAPS synthesis to the plastids (compare Figure 1). Therefore, we analysed a double mutant *fou8 apk3*, in which the only cytosolic APS kinase is disrupted. However, no differences in levels of glucosinolates and desulfo-glucosinolates and in the mRNA levels for glucosinolate synthesis genes were detected between *fou8* and the double mutant (Figure 3). In addition, while the *fou8 apk1 apk2* mutant lost many phenotypes visible on *fou8* [10], the *fou8 apk3* mutant was indistinguishable from *fou8* (Figure S1).

### Interaction of *FIERY1* with primary sulfate assimilation

We next asked whether the strong interconnection of primary and secondary sulfur metabolism [20], [22], [23], [24] can also be observed in the *fou8* mutant. Indeed, the transcript levels for most genes involved in sulfate reduction were found to differ significantly between Col-0 and *fou8* (Table 2). Whereas the mRNA levels of *APR* isoforms were higher in *fou8* leaves, the transcripts for *ATPS1* and *ATPS4* were reduced. Interestingly, these genes are regulated in the same way in sulfate deficient plants [25], [26]. Therefore we also tested the expression levels of two genes, *At5g48850* (*sulfate deficiency-induced 1*; *Low Sulfur 2*, *LS2*) and *At5g26220* (*Low Sulfur 5, LS5*), that belong among genes with the highest degree of up-regulation upon sulfate starvation [25], as markers for sulfate starvation expression response. The mRNAs for these genes were increased in *fou8* similar to sulfate deficient plants (Table 2). These results are in line with multiple signalling defects described for other *fry1* alleles [8], [9], and particularly with recently reported symptoms of phosphate deficiency in the *fry1* mutant [11]. Therefore, we revisited the previously described microarray data obtained with various alleles of *fry1/fou8* [8], [9] and used iterative group analysis [27] to compare genes altered in expression in *fry1* alleles with sets of genes showing differential expression by different treatments from the AtGen Express data set. Interestingly, in all three *fry1* related microarray experiments, the miss-regulated genes showed significant overlap with the set of genes regulated by sulfate starvation (Table S2). Thus, disruption of *FIERY1* seems to trigger the same expression response as sulfate starvation.

**Table 2:** Relative expression of sulfate assimilation genes in *fou8*.

	Relative expression	P value
*ATPS1*	0.67±0.07	0.0058
*ATPS2*	1.07±0.09	0.22
*ATPS3*	1.15±0.09	0.049
*ATPS4*	0.16±0.04	0.00011
*APR1*	4.4±0.85	0.00027
*APR2*	1.81±0.21	0.0027
*APR3*	1.75±0.32	0.018
*APK1*	1.36±0.19	0.022
*APK2*	2.34±0.3	0.0005
*APK3*	2.28±0.24	0.00001
*APK4*	0.57±0.14	0.015
*LS2*	4.18±0.79	0.0015
*LS5*	2.25±0.93	0.049

Col-0 and *fou8* plants were grown for 5 weeks in controlled environment room. Total RNA was isolated from leaves and the transcript levels of genes involved in sulfur metabolism was determined by quantitative RT-PCR. In two independent experiments qRT-PCR reactions were performed in triplicate for each of the three independent biological samples. The values in Col-0 were set to 1 for all genes. Results of one experiment are presented as means ± SD from three pools of three individual plants. P-values obtained by Student's T-test are also given.

**Figure 4:**
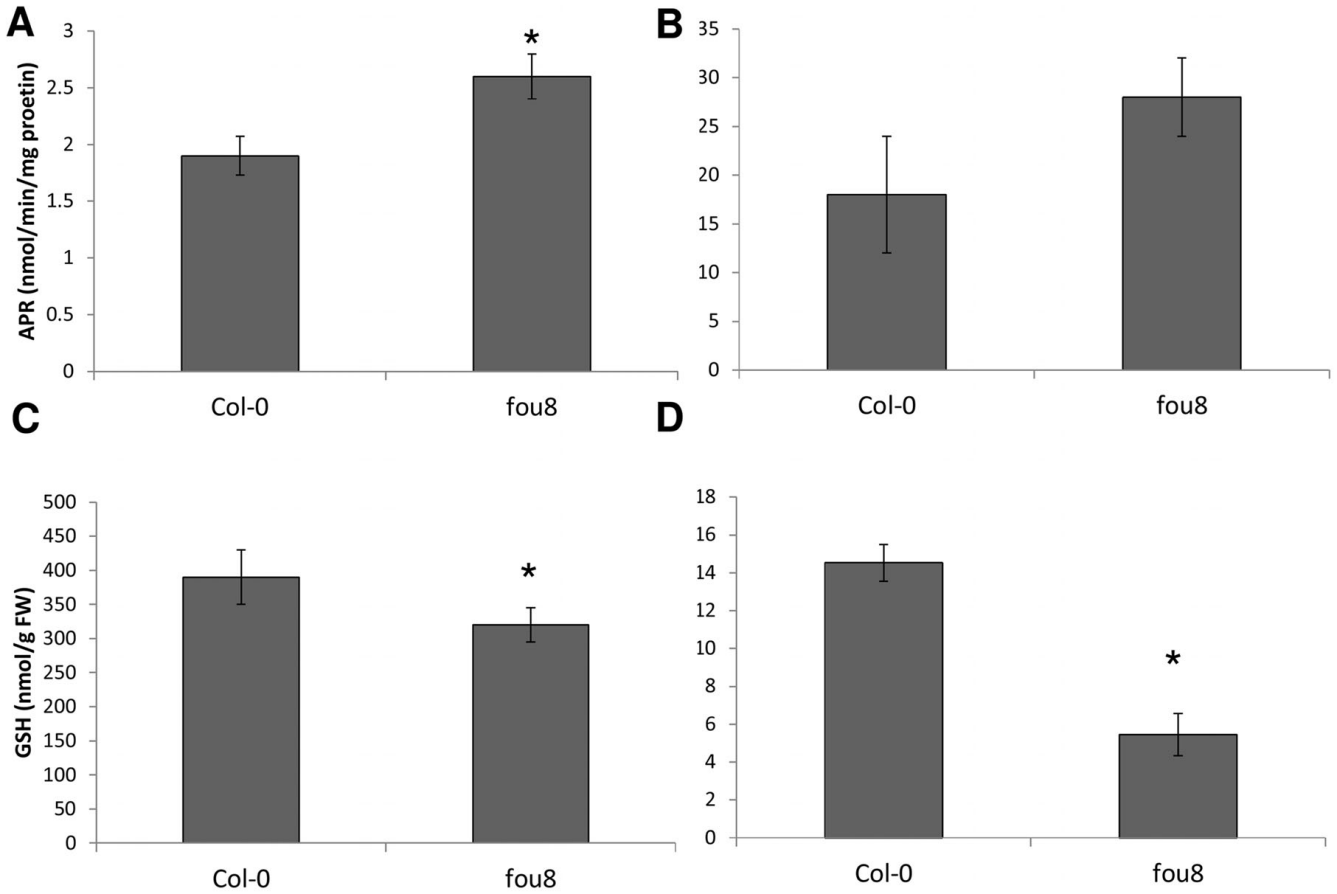
Accumulation of sulfur-containing compounds in *fou8*. Col-0 and *fou8* plants were grown for 5 weeks in controlled environment room. Leaves were harvested and **A** APR activity was determined. Levels of **B** cysteine **C** glutathione, and **D** sulfate were measured by HPLC. Results are presented as means ± SE from six individual plants, grown in two independent experiments. Asterisks mark values significantly different from Col-0 at P<0.05.

To test whether the sulfate starvation-like syndrome in *fou8* and other *fry1* alleles is restricted to gene expression or manifested also otherwise, we determined the enzyme activity of APR and the levels of sulfur containing metabolites. In agreement with the increased transcript levels, and consistent with response to sulfate starvation, APR activity was higher in leaves of *fou8* than in Col-0 (Figure 4a). While cysteine levels were unaffected, contents of the major thiol, glutathione, were lower in *fou8* leaves, again similar to plants under sulfate starvation (Figures 4b and 4c). Sulfate content was significantly lower in *fou8* and reached only ca. 50% of the levels in Col-0 (Figure 4d). Correspondingly, the total sulfur content in *fou8* leaves was significantly lower than in Col-0 (Table 3). Thus, the changes in expression pattern in *fou8* are not due to alteration in signalling but a genuine response to reduced levels of sulfate and sulfur containing metabolites.

**Table 3:** Mineral content of Col-0 and *fou8*.

	Col-0	*fou8*
K	5.8±0.1	3.5±0.1 *
Ca	5.5±0.3	5.6±0.1
Mg	2.1±0.1	2.2±0.1
P	1.9±0.1	2.1±0.1
S	1.2±0.1	0.96±0.08 *
Mn	0.034±0.005	0.04±0.004
Fe	0.014±0.002	0.021±0.013
Zn	0.0098±0.0024	0.0062±0.0009 *

Col-0 and *fou8* plants were grown for 30 days in soil in controlled environment room. Whole rosettes were harvested and the mineral levels were determined by X-ray fluorescence spectrophotometry as % of dry weight. Results from one of two independent experiments are presented as means ± SD from three individual plants. Values substantially different between the two genotypes (P<0.05) are marked by asterisks.

These results strongly indicate that it is not the external sulfate concentration that triggers sulfate deficiency response but instead the levels of internal sulfur-containing compounds. As Figure 4 shows, *fou8* possesses lower levels of sulfate and glutathione and both compounds may represent the sensed molecule. Indeed, reduction of glutathione content, e.g., by the inhibition of its synthesis by buthionine sulfoximine (BSO), induces APS reductase activity, similar to sulfate starvation [28]. We therefore asked whether reduced glutathione content in two independent Arabidopsis mutants in the first enzyme of glutathione synthesis, γ-glutamylcysteine synthetase, *cad2* and *rax1* [29], [30], also triggered sulfate starvation response. The iterative group analysis employed on microarray data from *cad2* and *rax1* mutants [30], however, did not show any overlap with sulfate deficiency response (Table S3).

**Figure 5:**
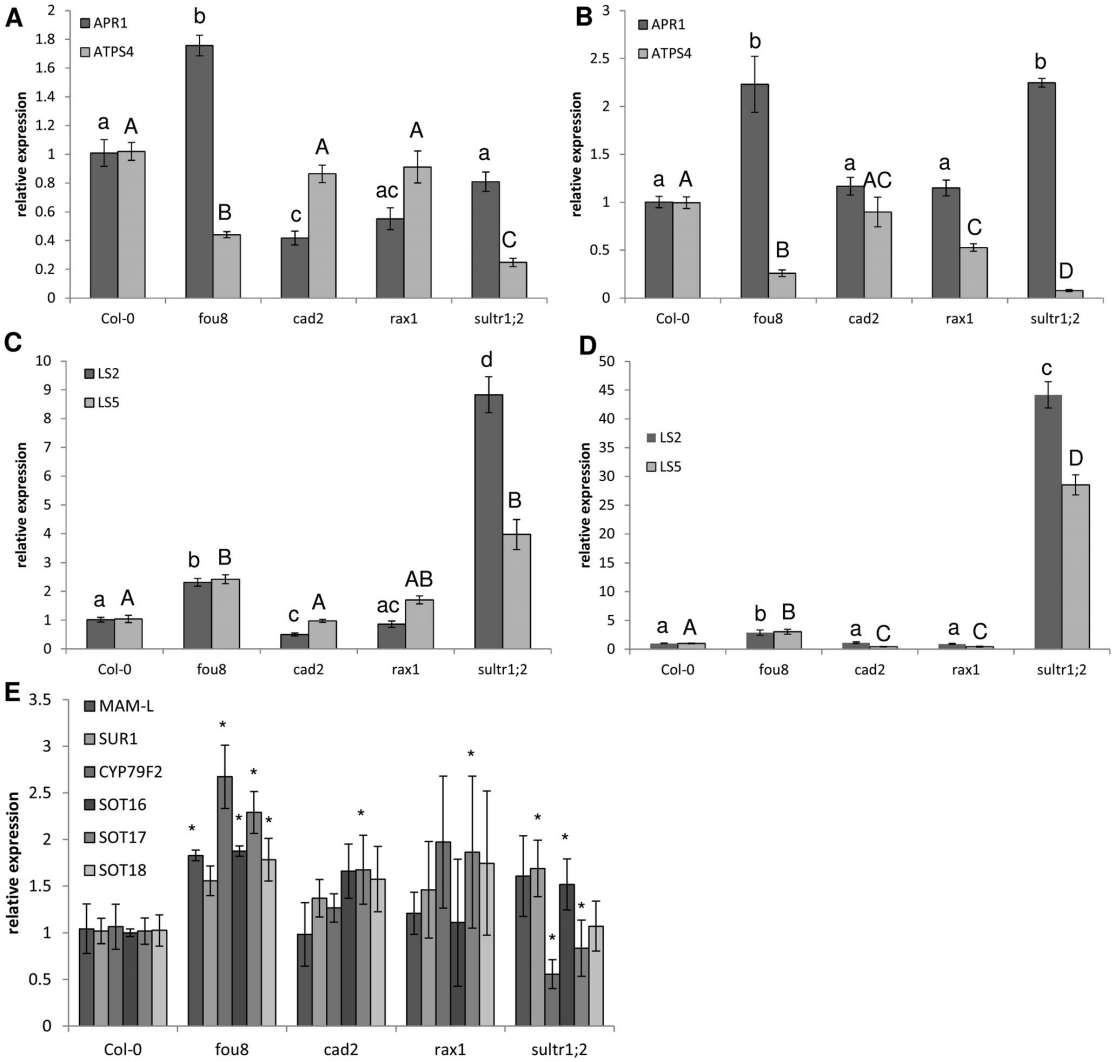
Comparison of mRNA levels of key genes of sulfur metabolism. Col-0, *fou8*, *cad2*, *rax1*, and *sultr1;2* plants were grown for 2.5 weeks on MS-agarose plates. Relative mRNA levels of *ATPS4* and *APR1* in **A** leaves and **B** roots and of *LS2* (At5g48850) and *LS5* (At5g26220) in **C** leaves and **D** roots and genes of glucosinolate synthesis in **E** leaves were determined. The qRT-PCR reactions were performed in triplicate, the values in Col-0 were set to 1 for all genes. Results are presented as means ± SE from six biological replicates from plants grown in two independent experiments. Different letters mark values significantly different at P<0.05 or in **E** asterisks show values significantly (P<0.05) different from Col-0.

To verify the transcriptomics data biochemically, we compared levels of sulfur-containing metabolites and gene expression of key markers of sulfate deficiency in 2-weeks old seedlings of *fou8*, *cad2*, *rax1*, and *sultr1;2* mutants. As expected, sulfate content was lower than in Col-0 in shoots and roots of *fou8* and *sultr1;2*, but not in *cad2* or *rax1*(Table 4). GSH levels were significantly lower in shoots and roots of *cad2* and *rax1* and also of *sultr1;2*. In leaves of *fou8* GSH levels were lower than in Col-0, while in roots GSH accumulated more than in the WT (Table 4). Glucosinolate levels were lower in *fou8*, as expected, and unaffected by mutations of γ-glutamylcysteine synthetase in *cad2* and *rax1*. The lower sulfate uptake capacity in *sultr1;2* resulted in a substantial reduction of glucosinolate accumulation, much greater than in the *fou8* mutant (Table 4). However, this reduction was not accompanied by accumulation of desulfo-glucosinolates in *sultr1;2* and these precursors were also at very low levels in *cad2* and *rax1* (Table 4). Transcript levels for *LS2*, *LS5*, and *APR1* were elevated and *ATPS4* was reduced in both leaves and roots of *fou8* compared to Col-0 (Figure 5), in agreement with the regulation of these genes by sulfate deficiency. On the other hand, in *cad2* and *rax1* the transcript levels of these genes were not affected or regulated in an opposite way than in sulfate deficient plants. The strong reduction in glucosinolate levels in *sultr1;2* did not cause similar co-ordinated up-regulation of the genes of glucosinolate synthesis as in *apk1 apk2* or *fou8* mutants (Figure 5e). Thus, diminishing glutathione content to ca. 15% WT levels does not trigger a sulfate deficiency response. In contrast, increased mRNA levels of *LS2*, *LS5*, and *APR1* correlate with reduced levels of internal sulfate in *fou8* and *sultr1;2*. Therefore, it seems that sulfate deficiency response in Arabidopsis is triggered by a reduction of internal sulfate levels.

**Table 4:** Contents of sulfur-containing metabolites in genotypes affected in sulfur metabolism.

		Sulfate (µmol/g FW)		Cysteine (nmol/g FW)	GSH (nmol/g FW)	Glucosinolates (µmol/g FW)	desulfo-glucosinolates (µmol/g FW)
shoots							
Col-0		7.8±0.9^a^		32±2^ a^	604±1^ a^	3.37±0.11^ a^	0.0014±0.002^ a^
*fou8*		3.8±0.5^ b^		31±11^ a^	575±7^ b^	1.89±0.08^ b^	3.3±0.4^ b^
*cad2*		9.4±0.2^ c^		167±34^ b^	96±8^ c^	3.24±0.16^ a^	0.074±0.016^ c^
*rax1*		8.2±1.3^ a^		64±6^ c^	216±25^ d^	3.63±0.33^ a^	0.026±0.02^ c^
*sultr1;2*		1.1±0.2^ d^		28±1^ a^	430±7^ e^	0.67±0.05^ c^	0.20±0.02^ d^
roots							
Col-0	5.1±0.1^ A^		170±11^ A^		457±26^ A^	n.d.	n.d.
*fou8*	4.3±0.1^ B^		198±7^ B^		571±23^ B^	n.d.	n.d.
*cad2*	6.1±0.1^ C^		289±45^ C^		90±15^ C^	n.d.	n.d.
*rax1*	7.0±0.5^ C^		233±30^ C^		160±20^ D^	n.d.	n.d.
*sultr1;2*	2.2±0.3^ D^		123±18^ D^		262±45^ E^	n.d.	n.d.

Col-0, *fou8*, *cad2*, *rax1*, and *sultr1;2* plants were grown for 2.5 weeks on MS-agarose plates. Sulfate, cysteine, glutathione in leaves and roots, and total glucosinolates and desulfo-glucosinolates levels in leaves were determined by HPLC. Results from one of two independent experiments are presented as means ± SD from three biological replicates, different letters mark values significantly different at P<0.05. n.d., not determined.

### Dissection of the Low Sulfur Phenotype

As the enzyme encoded by *FIERY1* has been shown to act on two types of substrates, adenosine bisphosphates as well as inositol bisphosphates, and as its mutation leads to defects in many signalling pathways, the low sulfur phenotype of *fou8* mutant can be caused by different mechanisms. To find out which of the *FIERY1* functions is responsible for the low accumulation of sulfur compounds, we analysed various mutants and transgenic lines related to *fou8*. To confirm that the observed metabolic changes are due to disruption of *FIERY1*, we used *fou8* complemented by the wild type allele (*fou8/FRY1*) [10]. In addition, the reduced levels of glucosinolates and sulfate and accumulation of desulfo-glucosinolates were confirmed in two T-DNA insertion lines in the *FIERY1* gene (Figure S2). As sulfate assimilation is highly responsive to jasmonate [31], [32], we tested another mutant with a similar fatty acid oxygenation phenotype connected with jasmonate accumulation, *fou2* [33], as well as the *aos* mutant deficient in jasmonate [34]. To test whether changes in accumulation of inositol related compounds might be responsible for the phenotype we analysed the *cvp2* mutant [35] that shares with *fou8* the defect in leaf venation pattern [9]. Since mutations in *FIERY1* affect processing of small RNAs and since sulfate assimilation is regulated by microRNA miR395 [36], we analysed a mutant in ribonucleases *xrn2 xrn3 xrn4* [11].

**Figure 6:**
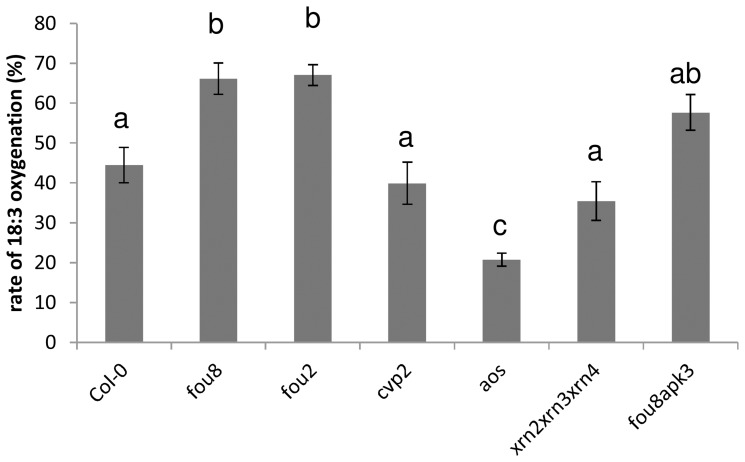
Variation in fatty acid oxygenation rate in *fou8* related genotypes. The various Arabidopsis lines were grown for 5 weeks in controlled environment room. Leaf juice was incubated for 2 min with 1-[^14^C]18∶3. Products were separated by thin layer chromatography and the radioactivity quantified. The rate of oxygenation is expressed as % of radioactivity in 18∶3-α-ketol. Results are presented as means ± SE from six individual plants grown in two independent experiments, different letters mark values significantly different at P<0.05.

First we checked the fatty acid oxygenation rates in these genotypes. Both *fou8* and *fou2*, which was isolated in the same screen as *fou8*, showed increased activity in this assay, whereas *aos* had an oxygenation rate lower than wild type. The other genotypes tested did not differ from Col-0 (Figure 6).

**Figure 7:**
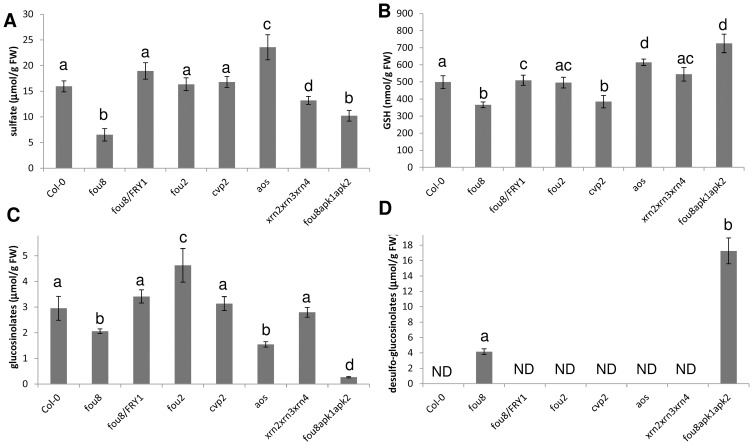
Accumulation of sulfur-containing compounds in *fou8* related genotypes. The various Arabidopsis lines were grown for 5 weeks in controlled environment room. Leaves were harvested and the levels of sulfur-containing compounds were determined by HPLC. **A** sulfate, **B** glutathione, **C** glucosinolates, and **D** desulfo-glucosinolates. Results are presented as means ± SE from six individual plants grown in two independent experiments with three replicates each. Different letters mark values significantly different at P<0.05; ND  =  not detectable.

The expression of the wild type *FIERY1* copy in *fou8* restored sulfate accumulation to the levels of Col-0 (Figure 7a). While there were no changes in sulfate levels in *cvp2* and *fou2*, sulfate accumulated to higher levels in *aos* and conversely, its content was lower in *xrn2 xrn3 xrn4* than in Col-0. Glutathione content, which was lower in *fou8*, was not completely restored to wild type levels by expression of *FIERY1*, however, the difference was much smaller than in the case of sulfate (Figure 7b). Interestingly, *aos* plants possessed higher GSH levels than other genotypes. Significant differences in glucosinolate levels were observed in mutants affected in jasmonate synthesis. Whereas *fou2* possessed higher levels of these metabolites than Col-0, *aos* contained significantly less glucosinolates than Col-0 and about the same as *fou8* (Figure 7c). However, these changes were not accompanied by accumulation of desulfo-glucosinolates, which were detected only in genotypes with disruption of *FIERY1* or *APK1* and *APK2* isoforms of APS kinase (Figure 7d).

**Figure 8:**
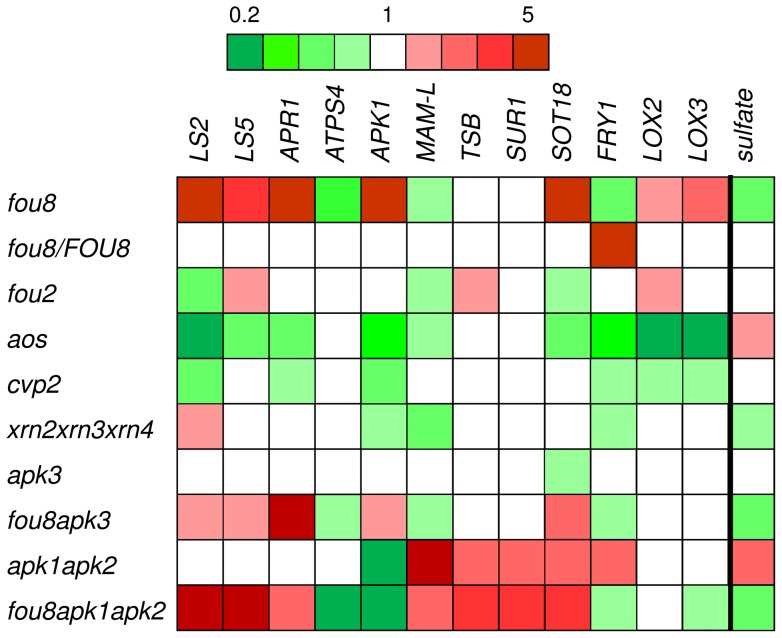
Expression analysis of Arabidopsis lines. The various Arabidopsis lines were grown for 5 weeks in controlled environment room. Total RNA was isolated from leaves and the transcript levels of genes involved in sulfur metabolism, glucosinolate synthesis, and jasmonate synthesis were determined by quantitative RT-PCR. The qRT-PCR reactions were performed in triplicate for each of the six independent biological samples from plants grown in two independent experiments. Results are presented as a heat map of relative mRNA levels compared to Col-0. For comparison, sulfate levels are presented in the same way on the far right.

**Figure 9:**
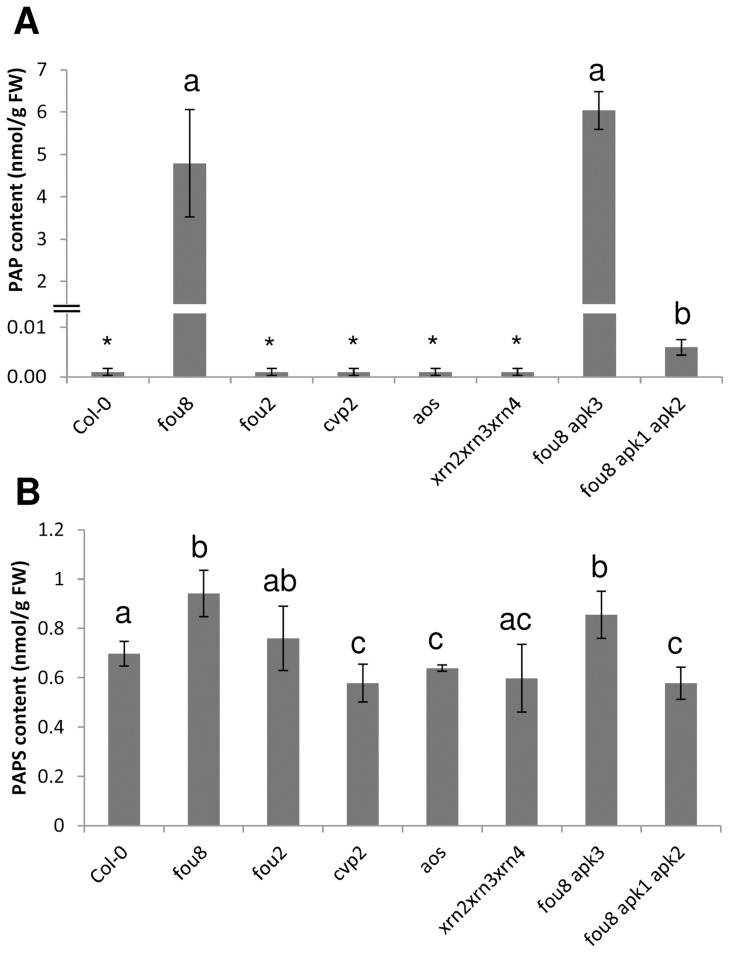
Accumulation of PAP and PAPS in *fou8* related genotypes. The various Arabidopsis lines were grown for 5 weeks in controlled environment room. Leaves were harvested and the levels of **A** PAP and **B** PAPS were determined by HPLC. Results are presented as means ± SD from four individual plants, grown in two independent experiments. Asterisks represents levels under detection limit, different letters mark values significantly different at P<0.05.

The expression profiles of key genes involved in sulfur, glucosinolate, and jasmonate metabolism were compared in these genotypes (Figure 8). Clearly, the sulfate deficiency expression pattern was retained in genotypes possessing the *fou8* mutation, as in these plants the sulfate levels did not differ from *fou8*. Also connected to the *fou8* mutation was the increased transcript level of *SOT18*, whereas other genes involved in glucosinolate synthesis were induced only in *fou8 apk1 apk2*, where the induction was driven by the *apk1 apk2* parent. The differences in glucosinolate levels between Col-0 and jasmonate affected genotypes *fou8*, *fou2*, and *aos* were not reflected in the transcript levels of the genes involved in glucosinolate synthesis. For example, mRNAs for *MAM-L* and *SOT18* were lower than in Col-0 in both *fou2* and *aos*, whereas the glucosinolate levels were increased in the former and lower in the latter (comp. Figure 7c). To find out whether the changes in sulfur metabolism are linked to PAP and/or PAPS accumulation we compared the levels of PAP and PAPS in these genotypes. Indeed, *fou8* mutation-containing genotypes accumulated PAP whereas this metabolite was not detectable in Col-0 or other lines analysed (Figure 9a). Similar levels of PAP were detected in *fou8 apk3* mutant, which is consistent with it being identical in appearance and sulfur metabolism to *fou8*. Reduced synthesis of PAPS in *fou8 apk1 apk2* mutants also prevented accumulation of PAP. The *fou8* mutant had higher levels not only of PAP but also of its precursor PAPS (Figure 9b). Disruption of cytosolic APS kinase in *fou8 apk3* plants did not affect the increased PAPS levels of *fou8*, while in *fou8 apk1 apk2* plants PAPS content was lower than in Col-0 (Figure 9b). Small changes in PAPS levels compared to Col-0 were observed in other genotypes, but they were much less pronounced than the alterations in PAP levels (Figure 9).

We also tested whether the alternative substrates of FIERY1, inositol polyphosphates, may be associated with the low sulfur phenotype. As the relevant inositol bis- and tri-phosphates are present in very low levels, as a surrogate we compared inositol (hexa)phosphate (IP6) levels in seeds. However, no differences were seen between *fou8* and Col-0 (data not shown), which is consistent with no differences in IP3 labelling between Col-0 and *fry1-6* [37].

**Figure 10:**
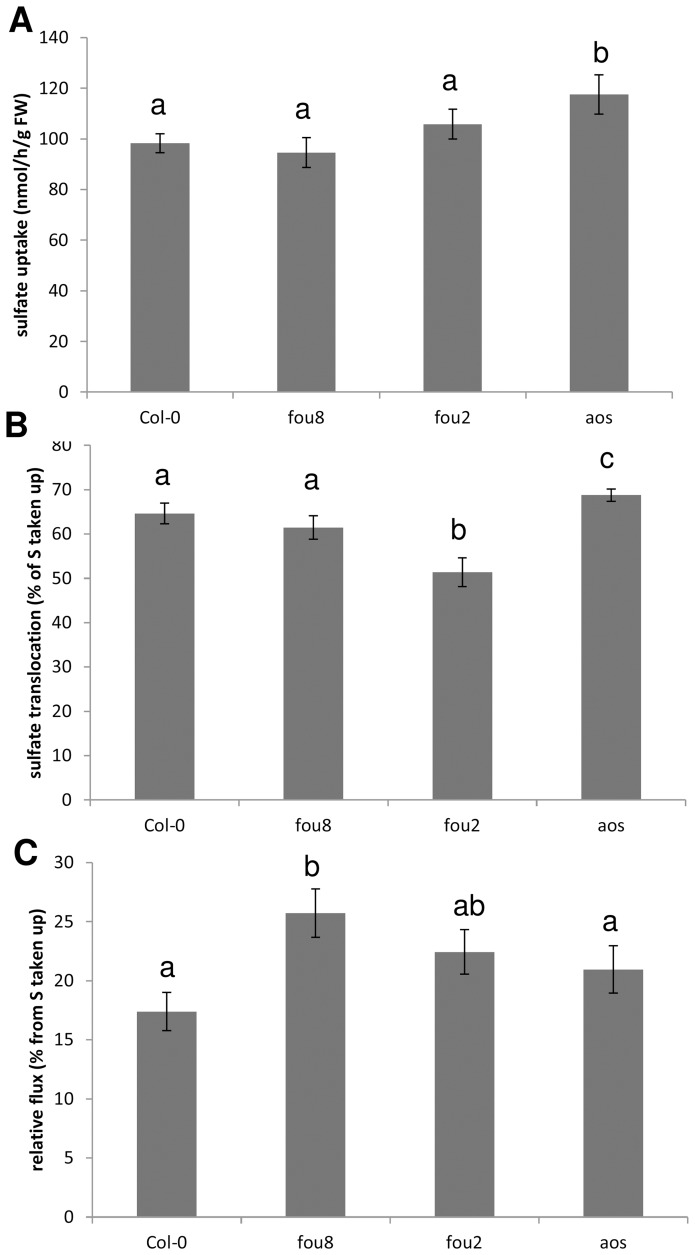
Sulfate uptake and flux through sulfate assimilation in *fou8* and related mutants. WT Col-0 and mutants *fou8*, *fou2*, and *aos* were grown for 3 weeks on MS-phytagel vertical plates in controlled environment room. The seedlings were incubated for four hours with their roots submerged in nutrient solution adjusted to sulfate concentration of 0.2 mM and supplemented with 6.7 μCi [^35^S]sulfate. Shoot and root material was harvested separately, and the flux was determined as incorporation of ^35^S from [^35^S] sulfate to thiols and proteins. **A** sulfate uptake, **B** Percentage of ^35^S transported to leaves from the [^35^S]sulfate taken up, **C** relative flux through the sulfate assimilation in the leaves calculated as % of incorporation in thiols and proteins from total [^35^S]sulfate taken up. Results are presented as means ± SE from six independent pools of 8 seedlings grown in two independent experiments. Values marked with an asterisk show significant (P≤0.05) difference from Col-0.

The sulfate content in *fou8* can be maintained lower than in Col-0 by reduced uptake or increased utilisation. Therefore we analysed flux through sulfate assimilation in genotypes differing in sulfate content (Figure 10). Sulfate uptake of three weeks old plants was not different between *fou8* and Col-0, however, it was somewhat increased in sulfate accumulating *aos* (Figure 10a). Also the sulfate translocation to shoots was higher in *aos* but identical in *fou8* and Col-0 and somewhat lower in *fou2* (Figure 10b). On the other hand, the flux through sulfate assimilation, determined as the percentage of ^35^S from the [^35^S]sulfate taken up incorporated into reduced compounds, thiols and proteins, was higher in *fou8* but identical in Col-0, *fou2*, and *aos* (Figure 10c). Thus, whereas in *aos* sulfate accumulates due to increased uptake and translocation to the leaves, the low sulfur phenotype in *fou8* seems not to be caused by differences in sulfate uptake but rather its reduction and utilisation.

## Discussion

### Links between FIERY1 and Glucosinolates

The *FIERY1* gene has been shown to affect a great range of cellular processes [2], [6], [7], [8], [9], [10], [11], [12], [37] and global analyses of transcriptome and metabolome of its mutants have been reported [8], [9]. It is however surprising that the direct metabolic effects of its disruption have been analysed only very recently, showing accumulation of PAP in the mutant [37]–[38]. We revealed the association of FIERY1 with glucosinolate metabolism (Figure 1 and 2) [20] and showed that, as expected, the glucosinolate levels are lower in this mutant than in wild type. The rationale for this expectation was the disruption of PAP removal in the mutant. Accumulation of PAP can be expected to directly inhibit sulfotransferases, as it will shift the reaction equilibrium towards the substrates, however, a direct biochemical evidence for this inhibition is not available. Alternatively, PAP may disrupt the transport of PAPS from plastids to the cytosol and reduce thus its concentration in the cytosol. Lower availability of PAPS in *apk1 apk2* mutants indeed resulted in low glucosinolate levels and accumulation of desulfo-glucosinolates [20]. Although a PAPS transporter has not yet been identified in plants, its presence in chloroplast envelope has been postulated from analyses of APS kinase mutants [20], [39]. To distinguish between the two options the cross between *fou8* and *apk3*, a mutant in the only cytosolic APS kinase, was analysed. If inhibition of PAPS transport by PAP caused the glucosinolate decrease in *fou8*, the phenotype in *fou8 apk3* plants should have been more severe. This was not the case, therefore, it seems that a direct inhibition of sulfotransferases by PAP is likely to be the cause for reduced glucosinolate content and accumulation of desulfo-glucosinolates. A compartment specific quantification of PAP and PAPS in the various combinations of *fou8* and *apk* mutants would unequivocally clarify the mechanisms, such measurements are, however, not technically feasible yet. This is supported by detecting increased transcript levels of all three desulfo-glucosinolate sulfotranferases (Figure 2 and 3). However, it has to be noted, that the levels of desulfo-glucosinolates were higher than would correspond to unused substrates, indicating an increased flux through the synthesis of glucosinolate precursors. This increase in flux was, however, much lower than that observed due to the disruption of PAPS synthesis in *apk1 apk2* mutants. In *apk1 apk2* almost all genes of glucosinolate synthesis network were co-ordinately up-regulated [20] but this was not the case in *fou8*. In various alleles of this mutant consistently only some genes of the network were induced, which is in accordance with the increased accumulation of desulfo-glucosinolates (Table S1). It is thus likely that the signal for the co-ordinated regulation of the network in *apk1 apk2* is not the desulfo-glucosinolates, as in such case the network would be induced in *fou8* to similar extent, unless the trigger is dependent on very high concentration of the precursors. It seems rather that a decrease in levels of one or more glucosinolates causes the up-regulation of the glucosinolate synthesis network. As aliphatic glucosinolates were reduced to much higher degree than indolic ones in *fou8*, it is possible that when one or more indolic glucosinolates decrease under certain level, a feedback mechanism induces the coordinated expression of the glucosinolate network. Indolic glucosinolates were indeed shown to exhibit negative feedback control of the pathway [40]. The mechanism of such positive feedback loop is unknown as direct evidence for regulation of gene expression by glucosinolates in plants is not available. Since the health promoting effects of glucosinolates is connected to stimulation of gene expression in human cells by glucosinolate degradation products [41], it can be expected that similar mechanisms exist in plants for example for the function of glucosinolates in immunity [15]–[16] [40]. In addition, the co-incidence of glucosinolate metabolic QTLs with eQTLs of the biosynthesis genes may also indicate an effect of glucosinolates on gene expression [42]. It is possible to speculate that such regulatory glucosinolate-related compound binds to transcription factor(s) and prevents their DNA binding. When the glucosinolate level is low, the inhibition is relieved and genes of the network are increasingly transcribed.

### Links of Jasmonate and Sulfur Metabolism

Jasmonate has a prominent role in coordinated regulation of sulfur metabolism [31], [32]. This is not surprising given the many functions of sulfur containing compounds in plant stress defense [43]. It has long been known that jasmonate signalling is important for regulation of glucosinolate synthesis, which is increased upon treatment with jasmonate [44], [45]. The induction is stronger for the indolic group of glucosinolates, which are considered to be more responsive to the environment [45]. Correspondingly, the indolic glucosinolates were much less affected in *fou8* than the aliphatic ones (Table 1), presumably since the inhibition of sulfotransferases was counteracted by the up-regulation of indolic glucosinolate synthesis due to increased jasmonate. Indeed, the glucosinolate content was increased in *fou2*, also with a larger contribution of the indolic group. However, the loss of jasmonate synthesis in *aos* mutant led to decreases of both groups of glucosinolates, so that there is at least some need for jasmonate for basal synthesis of aliphatic glucosinolates.

Another level of complexity in the interactions between glucosinolates, jasmonate, and sulfur metabolism has been added when potassium deficiency was shown to upregulate both jasmonate and glucosinolate synthesis [46] and when gene expression in *fou2* was found to be similar to that of potassium starved plants [47]. Interestingly, *fou8* plants possess significantly lower levels of potassium than Col-0 (Table 3). Thus, some of the changes in oxylipins and glucosinolates may actually be connected to reduced levels of potassium. The interactions between the mineral nutrients sulfate, phosphate, and potassium on one hand and metabolites such as jasmonate and glucosinolate on another hand are too complex to distinguish their causal relationships. It is possible that the low potassium is a consequence of low accumulation of anions sulfate and phosphate to keep the ionic balance but in the same way, the reduced accumulation of the anions may be secondary to the primary defect in potassium uptake. These findings thus open many exciting questions on the interconnection of plant mineral nutrition with primary and secondary metabolism and the *fou8* mutants may be an important tool in their dissection.

### Low Sulfur Phenotype of *fou8*

The effect of disruption of *FIERY1* on sulfur metabolism, however, did not stop at the glucosinolates. The transcripts of genes involved in sulfate assimilation, *APR*, *ATPS1* and *ATPS4* were regulated in *fou8* in the same way as in plants under sulfate starvation (Table 2). As this expression pattern could be due to the close links between primary and secondary sulfur metabolism [22] it was important to confirm the findings in a non-biased way. The iterative group analysis of available data on three independent microarray experiments showed that indeed, there is significant similarity in changes of global transcriptomes of *fou8/fry1* mutant and sulfate starved plants. Because the *fou8* mutant possesses higher levels of jasmonate [10], this very intriguing result reinforces the links between regulation of sulfate assimilation and jasmonate signalling: finding of jasmonate synthesis genes among those up-regulated by sulfate starvation [25], [26] and coordinated regulation of sulfur assimilation genes by jasmonate [31], [32]. However, as the sulfur-containing compounds sulfate and glutathione accumulated to lower levels in *fou8* than in Col-0 (Figure 4), the mutant indeed suffers from a mild sulfur deficiency. The lower content of sulfur is not connected to sulfate uptake, which is identical in *fou8* and Col-0 (Figure 10). This was surprising, since sulfate uptake is normally induced in sulfur deficient plants [4]. It seems therefore, that only a subset of sulfate starvation responses is triggered in *fou8*.

The analysis of *fou8* thus gave an interesting hint about the nature of sulfur sensing. The induction of the sulfate starvation response has always been associated with the decrease of external sulfate availability [25], [26]. Here we show, however, that at least some components of the response react to a decrease in internal levels of sulfur containing compounds. Results with *fou8* alone cannot distinguish between decrease of sulfate or glutathione as responsible for the signal, as both compounds were reduced in the mutant. To distinguish between these candidates existing microarray data of plants with reduced glutathione levels were interrogated. Firstly, we utilised the data from Arabidopsis plants where depletion of glutathione content without affecting sulfate levels was achieved by treatment with BSO [48]. Genes up-regulated by BSO treatment were similar to genes both up-regulated and down-regulated by sulfate starvation, thus not showing the clear relation like *fry1* mutants and making glutathione an unlikely candidate for the signal. This conclusion was corroborated by analysis of microarray datasets from *cad2* and *rax1* mutants in γ-glutamylcysteine synthetase [30]. These mutants possess only about 25% of wild type glutathione levels and are thus affected in its content to much greater degree than *fou8*. Since no overlap in expression between sulfate-starved plants and *cad2* or *rax1* mutants was observed (Table S3) reduction in internal sulfate levels seems to be the trigger for sulfate starvation response of gene expression in *fou8*. The correlation of sulfate deficiency-like expression of *APR1*, *ATPS4*, and the two marker genes for sulfate deficiency with sulfate levels but not GSH levels in a variety of mutants (Figure 5), strongly corroborates this conclusion. The finding that the induction of low sulfur responsive genes occurs also at normal sulfate supply is an important step in the search for sulfate sensing mechanism in plants. Actually, data allowing the same conclusion has been presented before in transcriptome analysis of sulfate deficient plants alongside the *sel1-10* mutant of *SULTR1;2* [49], however, the authors did not discuss such signalling.

Interestingly, the analysis of *cad2*, *rax1*, and *sultr1;2* mutants revealed further links between primary and secondary sulfur metabolism. Surprisingly, disruption of glutathione synthesis in *cad2* and *rax1* did not affect glucosinolate levels, in contrast to an allelic mutant *pad2* [50]. This could be caused by different growth conditions but also by different residual levels of glutathione in the mutants. Despite no effect on total glucosinolate levels, some desulfo-glucosinolates were detected in the mutants and transcript levels for some genes of the glucosinolate synthesis were increased, due to the well-known effect of glutathione deficiency on expression of defense genes [30]. In the *sultr1;2* mutant, the low capacity to uptake sulfate was manifested not only by reduced sulfate and glutathione levels, but also by strongly reduced glucosinolate content. In this case, however, only a very small increase in desulfo-glucosinolates and mRNA levels of the biosynthesis genes was observed (Table 4, Figure 5). Presumably, the sulfate starvation response initiated in this mutant prevents the up-regulation of glucosinolate synthesis.

So what triggers the decrease in sulfur accumulation in *fou8*? Four metabolites, phosphoinositols, PAP, PAPS, or jasmonate, are the main suspects as the signalling molecules. The phosphoinositols are unlikely, as *cvp2* that accumulates these compounds and shares several phenotypes with *fou8* [35] is not affected in sulfur metabolism. In addition, Estavillo et al. [37] did not observe any changes in inositol phosphates in *fry1* or other related genotypes. Jasmonate is also unlikely, as no alterations in sulfate or glutathione content was detected in *fou2* mutant that was isolated in the same genetic screen as *fou8* and no correlation between the fatty acid oxygenation phenotype and glucosinolate levels was observed (Figure 6). PAP and PAPS both accumulate in *fou8* and other *fry1* alleles [37], [38]. Their levels are reduced by crossing with *apk1 apk2*, but not *apk3*. However, the sulfate levels do not seem to be correlated with PAP or PAPS content as they remain low in *fou8 apk1 apk2* despite a large reduction of PAP and restoration of wild type PAPS levels (Figure 9). The large transcriptome reprogramming of *apk1 apk2* plants results in redirecting sulfur flow from secondary to primary metabolism as evidenced by increased flux through sulfate reduction and accumulation of reduced sulfur compounds [20], [22]. Similarly, in *fou8* mutant increased APR activity (Figure 4) and increased flux through the pathway (Figure 8) have been detected. It is thus possible that any restoration of sulfate levels in the *fou8 apk1 apk2* mutant due to reduced levels of PAP or PAPS is counteracted by the increased reduction rate and the effective sulfate content remains low. Such PAP induced sulfate deficiency resembles the local phosphate starvation in roots of *fry1* [11].

In conclusion, this study revealed several new phenotypes caused by disruption of *FIERY1*, the decrease in glucosinolate content and in accumulation of sulfur and potassium. These newly observed links between sulfur metabolism and jasmonate and between potassium and FIERY1 open up new avenues of research to better understand the integration of plant nutrition and metabolism. The analysis of *fou8* indicated that decreases in indolic glucosinolate(s) may be responsible for feedback up-regulation of glucosinolate synthesis in *apk1 apk2* and *fou8* mutants. Most importantly, the data presented here show that sulfur starvation responsive gene expression is not linked to external sulfate concentration but correlates with a decrease in internal sulfate levels. This is thus the first step in elucidation the mechanism of how plants sense adequate levels of sulfur supply.

## Materials and Methods

### Plant material and growth conditions

In this study, *Arabidopsis thaliana* (ecotype Col-0) were used as wild type. The *fou8* mutant and *fou8 apk1 apk2* plants were described in [10]. The *cvp2* and *xrn2 xrn3 xrn4* seeds were provided by F. Carland, Yale University and E. Marin, CNRS Aix-Marseille, respectively. T-DNA lines disrupting *FIERY1* gene SALK_020882 and SALK_151367 were obtained from T. Gigolashvili, University of Cologne. Plants were grown for 5 weeks in controlled environment room under a short day 10-h-light/14-h-dark cycle at constant temperature of 22°C, 60% relative humidity, and light intensity of 160 µE s^−1^m^−2^. For elemental analyses plants were soil-grown (one seed per 7 cm diameter pot) with 12 h light (100 µE s^−1^m^−2^), 70% humidity; daytime temperature 22°C and nighttime temperature 18°C. For the sulfate uptake and flux analysis the plants were grown for three weeks on vertical plates with Murashige Skoog media without sucrose supplemented with 0.5% phytagel. The plates were placed in a controlled environment room at 20°C under 16 h light/8 h dark cycle. For each experiment at least two independent sets of plants were grown and analysed, each including *fou8* as a control.

### Glucosinolate and Desulfo-glucosinolate Analysis

Glucosinolates were extracted from 50 mg frozen leaf material. The extraction and quantification of intact glucosinolates followed the protocol described in [20]. Native desulfo-glucosinolates were determined from the same extracts. However, desulfo-glucosinolates do not bind to DEAE-Sephadex. The flow through from loading the extract onto DEAE-Sephadex columns and the following washing step with water were collected and combined and analysed by HPLC-UV as described for mature glucosinolates [20].

### Expression Analysis

To determine mRNA levels total RNA was isolated by standard phenol/chlorophorm and LiCl precipitation. First-strand cDNA was synthesized from 1 µg of total RNA using QuantiTect Reverse Transcription Kit (Qiagen, Crawley, UK), which includes a DNAse step to remove possible DNA contamination. Quantitative real-time RT-PCR (qPCR) was performed using gene-specific primers (Table S4) and the fluorescent intercalating dye SYBR Green (Applied Biosystems, Warrington, UK) as described in [51]. All quantifications were normalized to the *TIP41* gene. The RT-PCR reactions were performed in duplicate for each of the three independent samples.

### Enzyme Assays

APS reductase activity was determined as the production of [^35^S]sulfite, assayed as acid volatile radioactivity formed in the presence of [^35^S]APS and dithioerythritol as reductant [52]. The protein concentrations were determined with a Bio-Rad protein kit (Bio-Rad, Hemel Hempstead, UK) with bovine serum albumin as a standard.

A lipoxygenase activity assay employed to compare the fatty acid oxygenation ratios was based on the oxygenation of radiolabeled linoleic acid (18∶2) as described previously [53]. The rate of oxygenation has been calculated as % of radioactivity in 18∶3-α-ketol from total radioactivity of linoleic acid used.

### Measurements of Sulfur-Containing Compounds

Sulfate contents were determined by ion-exchange HPLC method as described in [54]. Cysteine and GSH were analysed by HPLC as described by [52] from 20–30 mg of plant material. PAPS and PAP were extracted from leaves of *Arabidopsis* plants according to [55]. The adenosine compounds were derivatized with chloroacetaldehyde and separated by HPLC as described in [56]. For elemental analysis plants were harvested on day 30. The rosettes were ground in liquid nitrogen, lyophilized and analysed with a Philips PW2400 X-ray fluorescence spectrophotometer.

### Iterative Group Analysis

Original microarray data for *fry1* alleles were obtained from [8] and [9] and those for *cad2* and *rax1* from [30]. The expression data were normalised according to the AtGenExpress recommendations using a global mean normalisation excluding the top and bottom 2% of the data. Fold-changes in expression levels between the *fry1* mutants and corresponding wild types were calculated from means of the three biological replicates. The resulting data were compared to publically available transcriptome data using iterative group analysis [27] to identify microarray experiments which resulted in similar sets of regulated genes.

### Determination of Flux Through Sulfate Assimilation

The flux through sulfate assimilation was measured as incorporation of ^35^S from [^35^S] sulfate to thiols and proteins essentially as described in [57] and [58]. Three week old plants were transferred into 48-well plates containing 1 mL of MS nutrient solution adjusted to sulfate concentration of 0.2 mM and supplemented with 5.6 μCi [^35^S]sulfate (Hartmann Analytic, Braunschweig, Germany) and incubated in light for 4 hours. After the incubation the seedlings were washed 3 times with 2 mL of non-radioactive nutrient solution, carefully blotted with paper tissue, weighed, transferred into 1.5 mL tubes, and frozen in liquid nitrogen. The quantification of ^35^S in different S-containing compounds was performed exactly as in [22].

### Statistical analysis

The results were analysed for variance by the Genstat software using significance level of P = 0.05. When the two independent experiments were treated as variable in two-way ANOVA, the variances within the experiments were not different and the genotype x experiment interactions were not significant. Where only 2 genotypes were compared Student's T-test was used. For the figures the two experiments were analysed together (n = 6) and the data are presented as means ± standard error, for tables one experiment with 3 biological replicates is shown.

## Supporting Information

Figure S1 **Phenotype of** ***fou8 apk3*** **mutant.**
(PDF)

Figure S2 **Glucosinolate and sulfate accumulation in different alleles of** ***fry1.***
(PDF)

Table S1 **Relative expression of genes involved in glucosinolate synthesis in microarrays of** ***apk1 apk2*** **mutant** [**20**] **and three alleles of** ***fou8*** **/** ***fry1*** **** [**8**]–[**9**] **.**
(PDF)

Table S2 **AtGen express treatments which produce similar changes to genes significantly affected in expression in** ***fry1/fou8*** **.**
(PDF)

Table S3 **AtGen express treatments which produce similar changes to genes significantly affected in expression in** ***cad2*** **and** ***rax1*** **.**
(PDF)

Table S4 **Primers used for qRT-PCR.**
(PDF)

## References

[1] Quintero FJ, Garciadeblás B, Rodríguez–Navarro A (1996). The SAL1 gene of Arabidopsis, encoding an enzyme with 3′(2′),5′-bisphosphate nucleotidase and inositol polyphosphate 1-phosphatase activities, increases salt tolerance in yeast.. Plant Cell.

[2] Xiong L, Lee BH, Ishitani M, Lee H, Zhang C (2001). *FIERY1* encoding an inositol polyphosphate 1-phosphatase is a negative regulator of abscisic acid and stress signaling in Arabidopsis.. Genes Dev.

[3] Gil-Mascarell R, López-Coronado JM, Bellés JM, Serrano R, Rodríguez PL (1999). The Arabidopsis HAL2-like gene family includes a novel sodium-sensitive phosphatase.. Plant J.

[4] Takahashi H, Kopriva S, Giordano M, Saito K, Hell R (2011). Sulfur Assimilation in Photosynthetic Organisms: Molecular Functions and Regulations of Transporters and Assimilatory Enzymes.. Annu Rev Plant Biol.

[5] Gläser H-U, Thomas D, Gaxiola R, Montrichard F, Surdin-Kerjan Y (1993). Salt tolerance and methionine biosynthesis in *Saccharomyces cerevisiae* involve a putative phosphatase gene.. EMBO J.

[6] Xiong L, Lee H, Huang R, Zhu JK (2004). A single amino acid substitution in the Arabidopsis FIERY1/HOS2 protein confers cold signaling specificity and lithium tolerance.. Plant J.

[7] Gy I, Gasciolli V, Lauressergues D, Morel JB, Gombert J (2007). Arabidopsis FIERY1, XRN2, and XRN3 are endogenous RNA silencing suppressors.. Plant Cell.

[8] Wilson PB, Estavillo GM, Field KJ, Pornsiriwong W, Carroll AJ (2009). The nucleotidase/phosphatase SAL1 is a negative regulator of drought tolerance in Arabidopsis.. Plant J.

[9] Robles P, Fleury D, Candela H, Cnops G, Alonso-Peral MM (2010). The *RON1/FRY1/SAL1* gene is required for leaf morphogenesis and venation patterning in Arabidopsis.. Plant Physiol.

[10] Rodríguez VM, Chételat A, Majcherczyk P, Farmer EE (2010). Chloroplastic phosphoadenosine phosphosulfate metabolism regulates basal levels of the prohormone jasmonic acid in Arabidopsis leaves.. Plant Physiol.

[11] Hirsch J, Misson J, Crisp PA, David P, Bayle V (2011). A novel fry1 allele reveals the existence of a mutant phenotype unrelated to 5′->3′ exoribonuclease (XRN) activities in *Arabidopsis thaliana* roots.. PLoS One.

[12] Chen H, Xiong L (2010). The bifunctional abiotic stress signalling regulator and endogenous RNA silencing suppressor FIERY1 is required for lateral root formation.. Plant Cell Environ.

[13] Fahey JW, Zalcmann AT, Talalay P (2001). The chemical diversity and distribution of glucosinolates and isothiocyanates among plants.. Phytochemistry.

[14] Halkier BA, Gershenzon J (2006). Biology and biochemistry of glucosinolates.. Annu Rev Plant Biol.

[15] Bednarek P, Pilewska-Bednarek M, Svatos A, Schneider B, Doubsky J (2009). A glucosinolate metabolism pathway in living plant cells mediates broad-spectrum antifungal defense.. Science.

[16] Fan J, Crooks C, Creissen G, Hill L, Fairhurst S (2011). *Pseudomonas sax* genes overcome aliphatic isothiocyanate-mediated non-host resistance in Arabidopsis.. Science.

[17] Underhill EW, Wetter LR, Chisholm MD (1973). Biosynthesis of glucosinolates.. Biochem Soc Symp.

[18] Piotrowski M, Schemenewitz A, Lopukhina A, Müller A, Janowitz T (2004). Desulfoglucosinolate sulfotransferases from *Arabidopsis thaliana* catalyze the final step in the biosynthesis of the glucosinolate core structure.. J Biol Chem.

[19] Klein M, Reichelt M, Gershenzon J, Papenbrock J (2006). The three desulfoglucosinolate sulfotransferase proteins in Arabidopsis have different substrate specificities and are differentially expressed.. FEBS J.

[20] Mugford SG, Yoshimoto N, Reichelt M, Wirtz M, Hill L (2009). Disruption of adenosine-5′-phosphosulfate kinase in Arabidopsis reduces levels of sulfated secondary metabolites.. Plant Cell.

[21] Sønderby IE, Geu-Flores F, Halkier BA (2010). Biosynthesis of glucosinolates–gene discovery and beyond.. Trends Plant Sci.

[22] Yatusevich R, Mugford SG, Matthewman C, Gigolashvili T, Frerigmann H (2010). Genes of primary sulfate assimilation are part of the glucosinolate biosynthetic network in *Arabidopsis thaliana*.. Plant J.

[23] Mugford SG, Lee B-R, Koprivova A, Matthewman C, Kopriva S (2011). Control of sulfur partitioning between primary and secondary metabolism.. Plant J.

[24] Malitsky S, Blum E, Less H, Venger I, Elbaz M (2008). The transcript and metabolite networks affected by the two clades of Arabidopsis glucosinolate biosynthesis regulators.. Plant Physiol.

[25] Hirai MY, Fujiwara T, Awazuhara M, Kimura T, Noji M (2003). Global expression profiling of sulfur-starved Arabidopsis by DNA macroarray reveals the role of O-acetyl-l-serine as a general regulator of gene expression in response to sulfur nutrition.. Plant J.

[26] Nikiforova V, Freitag J, Kempa S, Adamik M, Hesse H (2003). Transcriptome analysis of sulfur depletion in *Arabidopsis thaliana*: interlacing of biosynthetic pathways provides response specificity.. Plant J.

[27] Breitling R, Amtmann A, Herzyk P (2004). Iterative Group Analysis (iGA): A simple tool to enhance sensitivity and facilitate interpretation of microarray experiments.. BMC Bioinformatics.

[28] Hartmann T, Hönicke P, Wirtz M, Hell R, Rennenberg H (2004). Sulfate assimilation in poplars (*Populus tremula x P. alba*) overexpressing γ-glutamylcysteine synthetase in the cytosol.. J Exp Bot.

[29] Cobbett CS, May MJ, Howden R, Rolls B (1998). The glutathione-deficient, cadmium-sensitive mutant, *cad2-1*, of *Arabidopsis thaliana* is deficient in gamma-glutamylcysteine synthetase.. Plant J.

[30] Ball L, Accotto GP, Bechtold U, Creissen G, Funck D (2004). Evidence for a direct link between glutathione biosynthesis and stress defense gene expression in Arabidopsis.. Plant Cell.

[31] Harada E, Kusano T, Sano H (2000). Differential expression of genes encoding enzymes involved in sulfur assimilation pathways in response to wounding and jasmonate in *Arabidopsis thaliana*.. J Plant Physiol.

[32] Jost R, Altschmied L, Bloem E, Bogs J, Gershenzon J (2005). Expression profiling of metabolic genes in response to methyl jasmonate reveals regulation of genes of primary and secondary sulfur-related pathways in *Arabidopsis thaliana*.. Photosynth Res.

[33] Bonaventure G, Gfeller A, Proebsting WM, Hortensteiner S, Chetelat A (2007). A gain-of-function allele of TPC1 activates oxylipin biogenesis after leaf wounding in Arabidopsis.. Plant J.

[34] Park JH, Halitschke R, Kim HB, Baldwin IT, Feldmann KA (2002). A knock-out mutation in allene oxide synthase results in male sterility and defective wound signal transduction in Arabidopsis due to a block in jasmonic acid biosynthesis.. Plant J.

[35] Carland F, Nelson T (2009). CVP2- and CVL1-mediated phosphoinositide signaling as a regulator of the ARF GAP SFC/VAN3 in establishment of foliar vein patterns.. Plant J.

[36] Kawashima CG, Matthewman CA, Huang S, Lee B-R, Yoshimoto N (2011). Interplay of SLIM1 and miR395 in regulation of sulfate assimilation in Arabidopsis.. Plant J.

[37] Estavillo GM, Crisp PA, Pornsiriwong W, Wirtz M, Collinge D (2011). Evidence for a SAL1-PAP Chloroplast Retrograde Pathway That Functions in Drought and High Light Signaling in Arabidopsis.. Plant Cell.

[38] Chen H, Zhang B, Hicks LM, Xiong L (2011). A nucleotide metabolite controls stress-responsive gene expression and plant development.. PLoS One.

[39] Mugford SG, Matthewman CA, Hill L, Kopriva S (2010). Adenosine 5′ phosphosulfate kinase is essential for Arabidopsis viability.. FEBS Lett.

[40] Clay NK, Adio AM, Denoux C, Jander G, Ausubel FM (2009). Glucosinolate metabolites required for an Arabidopsis innate immune response.. Science.

[41] Traka M, Gasper AV, Smith JA, Hawkey CJ, Bao Y (2005). Transcriptome analysis of human colon Caco-2 cells exposed to sulforaphane.. J Nutr.

[42] Wentzell AM, Rowe HC, Hansen BG, Ticconi C, Halkier BA (2007). Linking metabolic QTLs with network and cis-eQTLs controlling biosynthetic pathways.. PLoS Genet.

[43] Rausch T, Wachter A (2005). Sulfur metabolism: a versatile platform for launching defence operations.. Trends Plant Sci.

[44] Brader G, Tas E, Palva ET (2001). Jasmonate-dependent induction of indole glucosinolates in Arabidopsis by culture filtrates of the nonspecific pathogen *Erwinia carotovora*.. Plant Physiol.

[45] Mikkelsen MD, Petersen BL, Glawischnig E, Jensen AB, Andreasson E (2003). Modulation of CYP79 genes and glucosinolate profiles in Arabidopsis by defense signaling pathways.. Plant Physiol.

[46] Troufflard S, Mullen W, Larson TR, Graham IA, Crozier A (2010). Potassium deficiency induces the biosynthesis of oxylipins and glucosinolates in *Arabidopsis thaliana*.. BMC Plant Biol.

[47] Bonaventure G, Gfeller A, Rodríguez VM, Armand F, Farmer EE (2007). The *fou2* gain-of-function allele and the wild-type allele of Two Pore Channel 1 contribute to different extents or by different mechanisms to defense gene expression in Arabidopsis.. Plant Cell Physiol.

[48] Koprivova A, Mugford ST, Kopriva S (2010). Arabidopsis root growth dependence on glutathione is linked to auxin transport.. Plant Cell Rep.

[49] Maruyama-Nakashita A, Inoue E, Watanabe-Takahashi A, Yamaya T, Takahashi H (2003). Transcriptome profiling of sulfur-responsive genes in Arabidopsis reveals global effects of sulfur nutrition on multiple metabolic pathways.. Plant Physiol.

[50] Schlaeppi K, Bodenhausen N, Buchala A, Mauch F, Reymond P (2008). The glutathione-deficient mutant *pad2-1* accumulates lower amounts of glucosinolates and is more susceptible to the insect herbivore *Spodoptera littoralis*.. Plant J.

[51] Lee B-R, Koprivova A, Kopriva S (2011). Role of HY5 in regulation of sulfate assimilation in Arabidopsis.. Plant J.

[52] Koprivova A, North KA, Kopriva S (2008). Complex signaling network in regulation of adenosine 5′-phosphosulfate reductase by salt stress in Arabidopsis roots.. Plant Physiol.

[53] Caldelari D, Farmer EE (1998). A rapid assay for the coupled cell free generation of oxylipins.. Phytochemistry.

[54] Scheerer U, Haensch R, Mendel RR, Kopriva S, Rennenberg H (2010). Sulphur flux through the sulphate assimilation pathway is differently controlled by adenosine 5′-phosphosulphate reductase under stress and in transgenic poplar plants overexpressing γ-ECS, SO or APR.. J Exp Bot.

[55] Wirtz M, Hell R (2003). Production of cysteine for bacterial and plant biotechnology: application of cysteine feedback-insensitive isoforms of serine acetyltransferase.. Amino Acids.

[56] Bürstenbinder K, Rzewuski G, Wirtz M, Hell R, Sauter M (2007). The role of methionine recycling for ethylene synthesis in Arabidopsis.. Plant J.

[57] Kopriva S, Muheim R, Koprivova A, Trachsel N, Catalano C (1999). Light regulation of assimilatory sulfate reduction in *Arabidopsis thaliana*.. Plant J.

[58] Vauclare P, Kopriva S, Fell D, Suter M, Sticher L (2002). Flux control of sulphate assimilation in *Arabidopsis thaliana*: adenosine 5′-phosphosulphate reductase is more susceptible to negative control by thiols than ATP sulphurylase.. Plant J.

