# Supplementary figures and images for: Effects of fou8/fry1 Mutation on Sulfur Metabolism: Is Decreased Internal Sulfate the Trigger of Sulfate Starvation Response?
Source: PLoS One. 2012 Jun 18;7(6):e39425. doi: 10.1371/journal.pone.0039425 (PMC3377649; doi:10.1371/journal.pone.0039425)

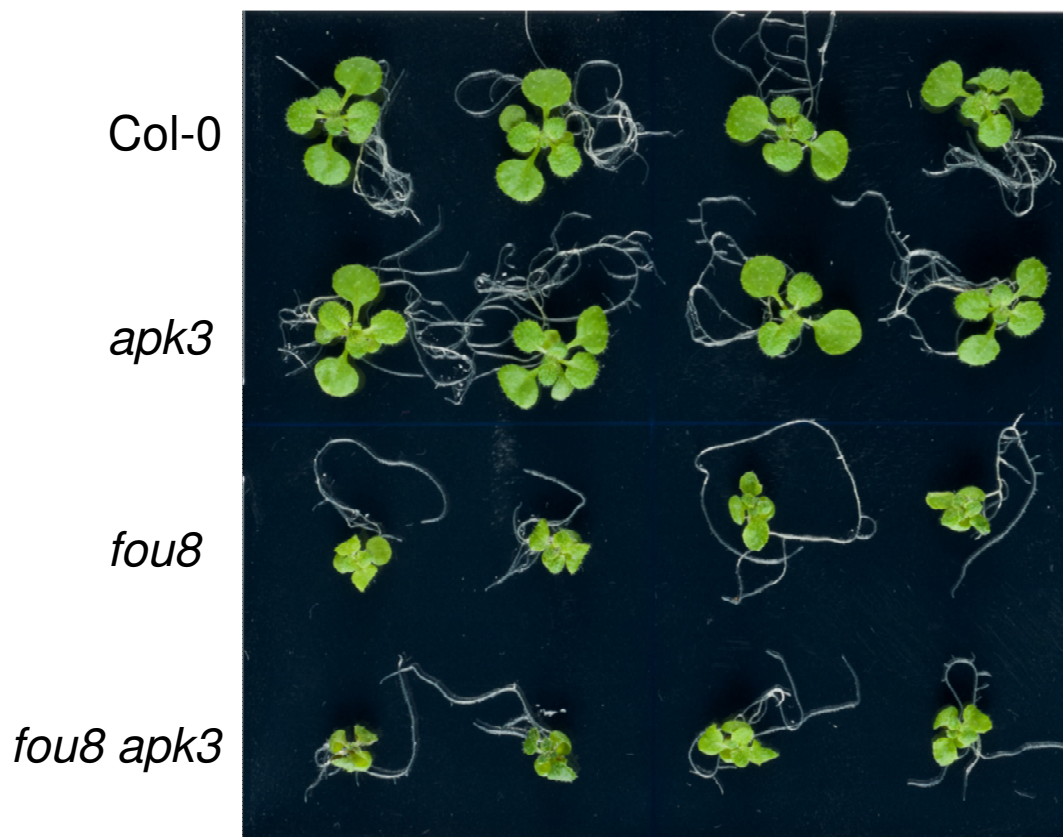

Supplemental Figure S1. Phenotypes of 2-weeks old seedlings of Col-0, *apk3*, *fou8*, and *fou8 apk3* plants.

Supplement: Figure S1 — Phenotype of fou8 apk3 mutant. (PDF) [file pone.0039425.s001.pdf]
