# Supplementary material for: Effects of fou8/fry1 Mutation on Sulfur Metabolism: Is Decreased Internal Sulfate the Trigger of Sulfate Starvation Response?
Source: PLoS One. 2012 Jun 18;7(6):e39425. doi: 10.1371/journal.pone.0039425 (PMC3377649; doi:10.1371/journal.pone.0039425)
Supplement: Figure S2 — Glucosinolate and sulfate accumulation in different alleles of fry1. (PDF) [file pone.0039425.s002.pdf]

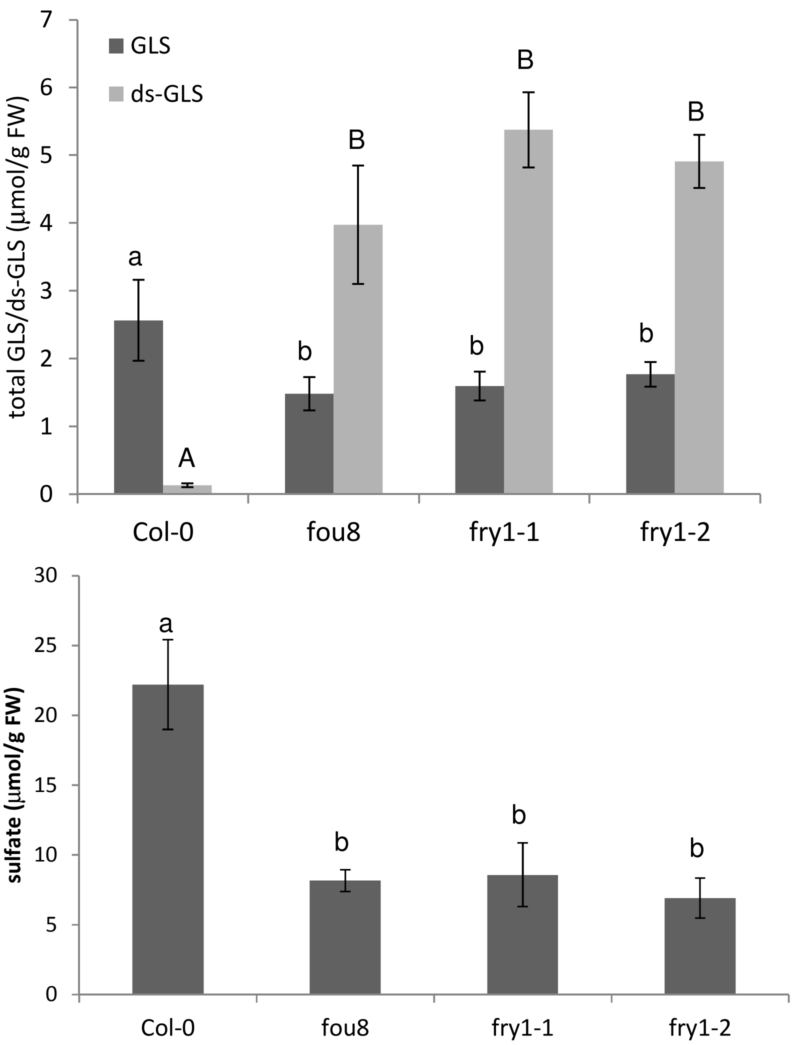

Supplemental Figure S2. Glucosinolate and sulfate accumulation in different alleles of *fry1.*

Col-0, *fou8*, and 2 T-DNA insertion lines in *FIERY1* gene (*fry1-1*, SALK_020882 and *fry1-2*, SALK_151367) plants were grown for 5 weeks in controlled environment room. (a) The total contents of glucosinolates and desulfo-glucosinolates, and (b) sulfate content were measured in leaves. Results are presented as means ± SD from three pools of three individual plants, different letters mark values significantly different at $P<0.05$.
