# Supplementary material for: Effects of fou8/fry1 Mutation on Sulfur Metabolism: Is Decreased Internal Sulfate the Trigger of Sulfate Starvation Response?
Source: PLoS One. 2012 Jun 18;7(6):e39425. doi: 10.1371/journal.pone.0039425 (PMC3377649; doi:10.1371/journal.pone.0039425)
Supplement: Table S1 — Relative expression of genes involved in glucosinolate synthesis in microarrays of apk1 apk2 mutant [20] and three alleles of fou8 / fry1 [8]–[9] . (PDF) [file pone.0039425.s003.pdf]

Supplemental Table S1. Relative expression of genes involved in glucosinolate synthesis in microarray analysis of *apk1 apk2* mutant [20] and three alleles of *fou8/fry1* [8] [9]. The complement of genes of glucosinolate synthesis network has been taken from [21]. Ratios of transcripts mutant vs. Col-0 and corresponding p-values (T-test) are presented as well as results of qPCR analysis with selected genes. Ratios greater than 1.5 are marked red and p-values smaller than 0.05 are yellow.

AGI		apk1 apk2		fry1/C24		alx8/Col-0		fry1-2/Col-0		qPCR	
AT3G19710	BCAT4	4.08	0.00010	1.24	0.1442	0.85	0.0615	1.25	0.0719		
AT4G12030	BAT5	2.79	0.00019	1.39	0.0995	0.59	0.0078	1.40	0.1123		
AT5G23010	MAM1	3.17	0.00000	1.39	0.0346	1.16	0.0480	1.23	0.2087		
AT5G23020	MAM3	10.11	0.00002	0.27	0.0173	0.35	0.0262	1.55	0.2073	0.8	0.087
AT4G13430	IPMI LSU	2.35	0.00009	1.36	0.0239	1.00	0.4655	1.21	0.0321		
AT2G43100	IPMI SSU2	4.76	0.00000	1.19	0.2378	1.15	0.0813	1.15	0.0380		
AT3G58990	IPMI SSU3	3.67	0.00001	0.56	0.0700	1.99	0.0039	1.10	0.1972		
AT5G14200	IPMDH1	3.25	0.00007	0.84	0.1864	1.10	0.1390	0.95	0.3279		
AT3G49680	BCAT3	1.76	0.00005	1.59	0.0080	1.03	0.3704	0.96	0.2935		
AT1G16410	CYP79F1	4.50	0.00005	1.39	0.1096	1.47	0.0260	1.04	0.4423		
AT1G16400	CYP79F2	4.50	0.00005	1.39	0.1096	1.47	0.0260	1.04	0.4423	4.1	0.004
AT4G13770	CYP83A1	2.19	0.00003	1.34	0.0482	1.23	0.0075	1.17	0.1770		
AT3G03190	GSTF11	3.39	0.00003	1.15	0.2200	1.04	0.4240	0.92	0.3084		
AT1G78370	GSTU20	3.38	0.00004	0.97	0.4444	0.81	0.0240	0.99	0.4761		
AT4G30530	GGP1	1.89	0.00067	1.69	0.0023	0.57	0.0008	1.17	0.1641		
AT2G20610	SUR1	1.93	0.00019	1.45	0.0540	1.43	0.0036	1.14	0.1032	0.82	0.174
AT2G31790	UGT74C1	2.09	0.00018	1.81	0.0070	1.26	0.0582	1.12	0.1653		
AT1G74090	SOT18	2.37	0.00020	1.44	0.0571	1.17	0.0595	1.23	0.0866	2.1	0.002
AT1G18590	SOT17	4.19	0.00001	1.56	0.0786	2.33	0.0017	1.11	0.3348	1.4	0.001
AT1G65860	FMO-GSOX1	3.25	0.00000	1.38	0.0950	0.75	0.0479	1.20	0.1401		
AT1G62540	FMO-GSOX2	2.03	0.01189	0.94	0.3468	1.00	0.4978	1.04	0.3799		
AT1G62560	FMO-GSOX3	3.12	0.00021	1.28	0.2105	0.78	0.0161	1.12	0.1429		
AT1G62570	FMO-GSOX4	1.21	0.04733	0.94	0.2450	1.39	0.1273	1.02	0.4076		
AT1G12140	FMO-GSOX5	1.22	0.00065	0.87	0.1586	1.33	0.0068	0.99	0.4645		
AT4G03050	AOP3	0.99	0.45940	0.92	0.2252	1.13	0.0044	1.32	0.0964		
AT4G03060	AOP2	3.23	0.00002	0.75	0.1390	1.86	0.0042	2.06	0.1109		
AT2G25450	GS-OH	2.12	0.00004	2.49	0.0186	1.89	0.0019	1.06	0.3372		
AT5G05260	CYP79A2	1.02	0.23466	0.96	0.3682	0.88	0.1200	1.03	0.2295		
AT4G39950	CYP79B2	8.99	0.00032	9.07	0.0066	8.76	0.0001	1.54	0.2270	21	0.0002
AT2G22330	CYP79B3	4.86	0.00037	5.10	0.0018	7.37	0.0001	2.26	0.1402		
AT4G31500	CYP83B1	2.02	0.00056	2.04	0.0068	2.43	0.0001	1.44	0.2152	1.8	0.02
AT2G30860	GSTF9	1.78	0.00002	1.28	0.0250	0.74	0.0002	0.93	0.0674		
AT1G24100	UGT74B1	2.00	0.00035	2.02	0.0462	1.87	0.0015	1.45	0.0860		
AT2G30870	GSTF10	1.30	0.00944	0.85	0.2100	1.44	0.0601	1.05	0.4239		

AT1G74100	SOT16	2.29	0.00210	1.27	0.1206	2.36	0.0017	1.34	0.0183	2.51	0.02
AT5G57220	CYP81F2	0.65	0.04030	0.41	0.0048	1.39	0.0057	1.16	0.1692		
AT5G61420	MYB28	1.33	0.00934	0.64	0.0133	0.56	0.0034	0.97	0.4146	1.12	0.073
AT5G07690	MYB29	2.17	0.00012	0.65	0.0936	0.85	0.1944	1.13	0.2963	1.62	0.0007
AT5G07700	MYB76	2.94	0.00376	1.16	0.1584	1.04	0.2749	0.87	0.2475		
AT1G18570	MYB51	1.45	0.02443	0.58	0.0794	1.06	0.4154	1.10	0.2044	0.6	0.02
AT1G74080	MYB122	1.02	0.43586	1.08	0.0376	0.88	0.0573	1.08	0.1921	12.9	0.02
AT5G60890	MYB34	2.21	0.00013	1.43	0.0868	2.95	0.0107	1.01	0.4755	6.8	0.0004
AT5G63980	FRY1	2.02	0.00014	0.99	0.4680	1.82	0.0832	0.93	0.3306	0.47	0.015
