# Supplementary material for: Effects of fou8/fry1 Mutation on Sulfur Metabolism: Is Decreased Internal Sulfate the Trigger of Sulfate Starvation Response?
Source: PLoS One. 2012 Jun 18;7(6):e39425. doi: 10.1371/journal.pone.0039425 (PMC3377649; doi:10.1371/journal.pone.0039425)
Supplement: Table S2 — AtGen express treatments which produce similar changes to genes significantly affected in expression in fry1/fou8 . (PDF) [file pone.0039425.s004.pdf]

Supplemental Table S2. AtGen express treatments which produce similar changes to genes significantly affected in expression in *fry1/fou8*.

Group number	Group Name	Total Members	Number Changed	P-Value
	**groups upregulated in ron1 mutant (Robles et al., 2010)**			
41	COLD ROOT 0.5-3HRS UP	91	5	2.96E-05
255	1 MIN RED LIGHT 45 MINS DARK UP	83	5	7.07E-05
253	FAR RED LIGTH 45 MINS UP	89	5	9.87E-05
257	RED LIGHT 45 MINS UP	96	5	0.000141
24	ABA DOWN	54	3	0.000632
70	GENOTOXIC SHOOT 0.5-3HRS DOWN	25	9	0.000813
68	GENOTOXIC ROOT 0.5-3HRS UP	121	2	0.001088
153	BRASSINAZOLE 220 10uM 3-12HRS UP	40	3	0.001122
128	WOUNDING SHOOT 0.25-3HRS DOWN	26	10	0.001462
222	SAR TISSUE FROM UNINFILTRATED HALF OF HALF PSEUDOMONAS SYRINGAE ES4326 INFILTRATED LEAF 4-16HRS UP	59	57	0.00195
227	SAR TISSUE FROM UNINFILTRATED HALF OF HALF PSEUDOMONAS SYRINGAE ES4326 INFILTRATED LEAF 1-2DAYS DOWN	63	61	0.002049
44	COLD SHOOT 0.5-3HRS DOWN	59	56	0.002061
188	ERYSIPHE ORONTII INFECTION 1-2DAYS DOWN	56	10	0.002167
106	OXIDATIVE SHOOT 0.5-3HRS DOWN	17	9	0.002206
112	SALT SHOOT 0.5-3HRS DOWN	71	70	0.002325
261	WHITE LIGHT 45 MINS UP	108	4	0.002523
91	METHYL JASMONATE DOWN	66	18	0.002567
129	WOUNDING ROOT 6-24HRS UP	80	13	0.002713
55	DROUGHT SHOOT 6-24HRS UP	84	27	0.002733
95	OSMOTIC SHOOT 0.5-3HRS DOWN	86	85	0.002798
229	**SULPHATE STARVATION 12-24HRS UP**	16	3	0.00303
120	UV-B SHOOT 0.25-3HRS DOWN	96	92	0.003124
117	UV-B ROOT 0.25-3HRS UP	98	21	0.003189
	**groups upregulated in fry1 mutant (Wilson et al., 2009)**			
75	GENOTOXIC SHOOT 6-24HRS DOWN	22	14	0.000733
229	**SULPHATE STARVATION 12-24HRS UP**	16	7	0.000736
55	DROUGHT SHOOT 6-24HRS UP	84	3	0.000983
225	SAR TISSUE FROM UNINFILTRATED HALF OF HALF PSEUDOMONAS SYRINGAE ES4326 AVRRPT2 INFILTRATED LEAF 1-2DAYS DOWN	23	23	0.001092
186	ERYSIPHE ORONTII INFECTION 6-18HRS UP	20	3	0.001392
102	OXIDATIVE ROOT 6-24HRS UP	44	9	0.001533
129	WOUNDING ROOT 6-24HRS UP	80	2	0.001703
153	BRASSINAZOLE 220 10uM 3-12HRS UP	40	5	0.001706
121	UV-B ROOT 6-24HRS UP	33	8	0.001728
222	SAR TISSUE FROM UNINFILTRATED HALF OF HALF PSEUDOMONAS SYRINGAE ES4326 INFILTRATED LEAF 4-16HRS UP	59	22	0.001919
44	COLD SHOOT 0.5-3HRS DOWN	59	55	0.001963
227	SAR TISSUE FROM UNINFILTRATED HALF OF HALF PSEUDOMONAS SYRINGAE ES4326 INFILTRATED LEAF 1-2DAYS DOWN	63	61	0.002049

218	PROTEASOME INHIBITOR MG132 10uM 3HRS UP	94	4	0.002144
112	SALT SHOOT 0.5-3HRS DOWN	71	70	0.002324
53	DROUGHT ROOT 6-24HRS UP	48	12	0.002498
58	HAIRPINZ DOWN	80	78	0.002602
255	1 MIN RED LIGHT 45 MINS DARK UP	83	81	0.002708
95	OSMOTIC SHOOT 0.5-3HRS DOWN	86	80	0.002798
253	FAR RED LIGTH 45 MINS UP	89	50	0.002901
120	UV-B SHOOT 0.25-3HRS DOWN	96	90	0.003124
257	RED LIGHT 45 MINS UP	96	93	0.003129
117	UV-B ROOT 0.25-3HRS UP	98	25	0.003189
188	ERYSIPHE ORONTII INFECTION 1-2DAYS DOWN	56	49	0.003529
	**groups upregulated in alx8 mutant (Wilson et al., 2009)**			
222	SAR TISSUE FROM UNINFILTRATED HALF OF HALF PSEUDOMONAS SYRINGAE ES4326 INFILTRATED LEAF 4-16HRS UP	59	8	1.63E-09
55	DROUGHT SHOOT 6-24HRS UP	84	7	4.60E-07
117	UV-B ROOT 0.25-3HRS UP	98	6	1.78E-05
129	WOUNDING ROOT 6-24HRS UP	80	5	2.01E-05
218	PROTEASOME INHIBITOR MG132 10uM 3HRS UP	94	3	0.000168
61	LIPOPOLYSACCARIDE UP	25	3	0.00021
53	DROUGHT ROOT 6-24HRS UP	48	2	0.000338
191	ERYSIPHE ORONTII INFECTION 3-5DAYS DOWN	102	3	0.000402
228	**SULPHATE STARVATION 2-8HRS UP**	5	5	0.00045
229	**SULPHATE STARVATION 12-24HRS UP**	16	6	0.000541
75	GENOTOXIC SHOOT 6-24HRS DOWN	22	11	0.000717
77	HEAT ROOT 0.25-3HRS DOWN	42	3	0.000734
186	ERYSIPHE ORONTII INFECTION 6-18HRS UP	20	5	0.000919
159	6-DEOXOCATHASTERONE (1 UM, 3 HOURS) DOWN	116	5	0.001144
102	OXIDATIVE ROOT 6-24HRS UP	44	2	0.001187
35	BRASSINOLIDE UP	41	5	0.001525
70	GENOTOXIC SHOOT 0.5-3HRS DOWN	25	6	0.001584
227	SAR TISSUE FROM UNINFILTRATED HALF OF HALF PSEUDOMONAS SYRINGAE ES4326 INFILTRATED LEAF 1-2DAYS DOWN	63	61	0.002049
44	COLD SHOOT 0.5-3HRS DOWN	59	56	0.002067
112	SALT SHOOT 0.5-3HRS DOWN	71	69	0.002379
194	EHTYLENE INHIBITOR AMINOETHOXYVINYLGLYCINE 10uM 3HRS UP	117	4	0.002492
58	HAIRPINZ DOWN	80	78	0.002602
255	1 MIN RED LIGHT 45 MINS DARK UP	83	77	0.002702
188	ERYSIPHE ORONTII INFECTION 1-2DAYS DOWN	56	6	0.002774
95	OSMOTIC SHOOT 0.5-3HRS DOWN	86	80	0.002798
156	BRASSINAZOLE 91 10uM 3-12HRS DOWN	66	7	0.002857
253	FAR RED LIGTH 45 MINS UP	89	86	0.002904
200	GA BIOSYTH INHIBITOR UNICONAZOLE (10 UM, 3-12 HOURS) UP	59	6	0.002966
120	UV-B SHOOT 0.25-3HRS DOWN	96	95	0.003124
257	RED LIGHT 45 MINS UP	96	93	0.003126
153	BRASSINAZOLE 220 10uM 3-12HRS UP	40	7	0.003284
189	ERYSIPHE ORONTII INFECTION 6-18HRS DOWN	15	3	0.003691
91	METHYL JASMONATE DOWN	66	60	0.00371
24	ABA DOWN	54	2	0.003721
