# Supplementary material for: Effects of fou8/fry1 Mutation on Sulfur Metabolism: Is Decreased Internal Sulfate the Trigger of Sulfate Starvation Response?
Source: PLoS One. 2012 Jun 18;7(6):e39425. doi: 10.1371/journal.pone.0039425 (PMC3377649; doi:10.1371/journal.pone.0039425)
Supplement: Table S3 — AtGen express treatments which produce similar changes to genes significantly affected in expression in cad2 and rax1 . (PDF) [file pone.0039425.s005.pdf]

Supplemental Table S3. AtGen express treatments which produce similar changes to genes significantly affected in expression in *cad2* and *rax1* (Ball *et al.,* 2004).

Group number	Group Name	Total Members	Number Changed	P-Value
	**groups upregulated in *cad2* mutant**			
41	COLD ROOT 0.5-3HRS UP	22	6	1.61E-10
166	3-DEHYDRO-6-DEOXOTEASTERONE (1 UM, 3 HOURS) UP	40	6	2.34E-10
168	3-DEHYDROTEASTERONE (1 UM, 3 HOURS) UP	69	8	5.76E-10
172	TYPHASTEROL (1 UM, 3 HOURS) UP	76	8	7.16E-10
79	HEAT SHOOT 0.25-3HRS DOWN	59	11	1.28E-09
178	BRASSINOLIDE (1 UM, 3 HOURS) UP	92	8	1.34E-09
49	DROUGHT ROOT 0.25-3HRS UP	33	8	1.63E-09
174	6-DEOXOCASTASTERONE (1 UM, 3 HOURS) UP	84	13	2.25E-09
176	CASTASTERONE (100 NM, 3 HOURS) UP	91	13	2.58E-09
43	COLD SHOOT 0.5-3HRS UP	55	12	2.79E-09
164	TEASTERONE (1 UM, 3 HOURS) UP	38	5	6.94E-09
112	SALT SHOOT 0.5-3HRS DOWN	34	9	7.19E-09
170	6-DEOXOTYPHASTEROL (1 UM, 3 HOURS) UP	32	7	1.95E-08
162	6-DEOXOTEASTERONE (1 UM, 3 HOURS) UP	24	4	3.77E-08
74	GENOTOXIC SHOOT 6-24HRS UP	58	7	8.62E-08
100	OXIDATIVE SHOOT 6-24HRS UP	61	7	1.23E-07
88	AUXIN UP	41	7	1.59E-07
160	CATHASTERONE (1 UM, 3 HOURS) UP	12	3	7.30E-07
51	DROUGHT SHOOT 0.25-3HRS UP	76	9	2.12E-05
158	6-DEOXOCATHASTERONE (1 UM, 3 HOURS) UP	12	3	2.92E-05
72	GENOTOXIC ROOT 6-24HRS UP	83	8	3.12E-05
35	BRASSINOLIDE UP	13	2	4.31E-05
188	ERYSIPHE ORONTII INFECTION 1-2DAYS DOWN	22	4	6.92E-05
189	ERYSIPHE ORONTII INFECTION 6-18HRS DOWN	5	3	0.000701
138	24HRS 3uM ABA DURING 48HRS SEED IMBIBITION DOWN	44	5	0.001247
70	GENOTOXIC SHOOT 0.5-3HRS DOWN	12	10	0.001528
95	OSMOTIC SHOOT 0.5-3HRS DOWN	32	4	0.001756
149	AUXIN INHIBITOR UP (BOTH PCIB & 2,4,6-t)	20	2	0.002579
60	FLAGELLIN DOWN	19	18	0.003737
	**groups upregulated in *rax1* mutant**			
144	2,4,6-t AUXIN INHIBITOR DOWN	69	8	2.53E-05
209	JASMONIC ACID BIOSYNTH INHIBITOR IBUPROFEN 10uM 3HRS DOWN	75	8	0.000165
169	3-DEHYDROTEASTERONE (1 UM, 3 HOURS) DOWN	55	4	0.001534
64	GIBBERELLIN UP	4	3	0.001551
152	AUXIN TRANSPORT INHIBITOR DOWN (BOTH NPA & TIBA)	62	6	0.001687
75	GENOTOXIC SHOOT 6-24HRS DOWN	11	9	0.001898
73	GENOTOXIC ROOT 6-24HRS DOWN	13	4	0.001899
189	ERYSIPHE ORONTII INFECTION 6-18HRS DOWN	5	4	0.002337
199	GA BIOSYTH INHIBITOR PROPICONAZOLE (10 UM, 3-12 HOURS) DOWN	22	11	0.002543
203	GA BIOSYTH INHIBITOR PACLOBUTRAZOL (10 UM, 3-12 HOURS) DOWN	37	4	0.002753
70	GENOTOXIC SHOOT 0.5-3HRS DOWN	12	8	0.002785
60	FLAGELLIN DOWN	19	11	0.002851
162	6-DEOXOTEASTERONE (1 UM, 3 HOURS) UP	24	16	0.003246

95	OSMOTIC SHOOT 0.5-3HRS DOWN	32	29	0.003584
112	SALT SHOOT 0.5-3HRS DOWN	34	28	0.003668
160	CATHASTERONE (1 UM, 3 HOURS) UP	12	8	0.00375
158	6-DEOXOCATHASTERONE (1 UM, 3 HOURS) UP	12	8	0.00375
272	RED LIGHT 4HRS DOWN	57	5	0.003782
