# Supplementary material for: Effects of fou8/fry1 Mutation on Sulfur Metabolism: Is Decreased Internal Sulfate the Trigger of Sulfate Starvation Response?
Source: PLoS One. 2012 Jun 18;7(6):e39425. doi: 10.1371/journal.pone.0039425 (PMC3377649; doi:10.1371/journal.pone.0039425)
Supplement: Table S4 — Primers used for qRT-PCR. (PDF) [file pone.0039425.s006.pdf]

Supplemental Table S4. Primers used for qRT-PCR.

gene	5' primer	3' primer
TIP41	GTGAAAACTGTTGGAGAGAAGCA	TCAACTGGATACCCTTTCGC
ATPS1	CACTCGGAGGTTTCATGAGAG	AGACGTAGCGAGTTAAAATGAAGAG
ATPS2	GATCTTGAGTGGGTTCATGTGAT	CTCATCTTCTCTCATGAACCCTTT
ATPS3	TGGGTTTATGAGGGAATCTGAG	GACCCATCATCGAGATTCAAC
ATPS4	CAAAGGTTTCATGAGACAGTCAG	GAGCCGGAACGAGTTAAAATG
APR1	CGATCAAGTATCCGTCGTAGAAG	GGACAAGATTCAAGAACGAAGTC
APR2	AAAAGAGCTCCACGGGCTAT	CGACATGAGTGAATCAACATCTC
APR3	CCAATCAAGTATCCATCAGAGAAG	CCGAACAAGATTCAAGAAAGATG
APK1	CCTTACGAGCCACCATTGAACTG	GCCATTTCGATAGGAGAAGTTCCT
APK2	CAAAATCAAAGGCTTCACTGGAATC	TGTTTCAGCACTACCTCGCAATT
APK3	GTTGAAAGAGAAAGAGGGAGAGTGTC	AGAGATCACTTCCTCAGCCATAGC
APK4	TCTTCTTCTCTGTGTGAAATGGCAG	TTCTTCAGGTATCCATTTTGGTCC
SULTR2;1	CAGAGAGTTTTGAATCTCTCTCACATC	CCATCTGGATCATGTGTGTTG
LS5 (At5g26220)	TCCACCGGAGCTATTTGC	CGTTCCAGTACTCCATTGCT
LS2 (At5g48850)	TCCCTGTGGAGACACTCCTT	CCATCTCCGGGTTCTTCTCT
SOT17	GGAATCCAAAACCATAAACGACG	CGGATCTTTTGGTCTCCAGCC
SOT18	CCCTACCGAGTCACGACGAGA	GGTAGCCACCAGTAACCACCATACT
MAM-L	TTCGTTACTTCTCACATCGTCGAG	GGAGCAACATGAGACGAACAGAGT
SUR1	GGATTCTCCCCGCAAGAC	CATCCAGCGGTCAGAAAA
TSB	ACGAAGAAGCGTTGGAAGC	GGTAAGCTAGTGCGTGTGAGG
LOX2	CTTACCCGCGGATCTCATC	ACTCCATGTTCTGCGGTCTT
LOX3	CGCCAATCAACAGTTTCTGA	CTCGTCTCGTGGCACATACA
FRY1	CTGAAGGTGGTCCAAATGGT	TGATCTCCCCTCAGAAATCC
